# Core–shell dry adhesives for rough surfaces via electrically responsive self-growing strategy

**DOI:** 10.1038/s41467-022-35436-6

**Published:** 2022-12-10

**Authors:** Hongmiao Tian, Duorui Wang, Yahui Zhang, Yuanze Jiang, Tianci Liu, Xiangming Li, Chunhui Wang, Xiaoliang Chen, Jinyou Shao

**Affiliations:** 1 Micro-and Nano-technology Research Center, State Key Laboratory for Manufacturing Systems Engineering, Xi’an Jiaotong University, Xi’an, Shaanxi 710049 China; 2 Frontier Institute of Science and Technology (FIST), Xi’an Jiaotong University, Xi’an, Shaanxi 710049 China

**Keywords:** Mechanical properties, Bioinspired materials, Polymers

## Abstract

Bioinspired dry adhesives have an extraordinary impact in the field of robotic manipulation and locomotion. However, there is a considerable difference between artificial structures and biological ones regarding surface adaptability, especially for rough surfaces. This can be attributed to their distinct structural configuration and forming mechanism. Here, we propose a core–shell adhesive structure that is obtained through a growth strategy, i.e., an electrically responsive self-growing core–shell structure. This growth strategy results in a specific mushroom-shaped structure with a rigid core and a soft shell, which exhibits excellent adhesion on typical target surfaces with roughness ranging from the nanoscale to the microscale up to dozens of micrometers. The proposed adhesion strategy extends dry adhesives from smooth surfaces to rough ones, especially for rough surfaces with roughness up to dozens or hundreds of micrometers, opening an avenue for the development of dry adhesive-based devices and systems.

## Abstract (web summary)

The design of bioinspired dry adhesives mimicking closely the structure and surface adaptability of biological adhesives is challenging. Here, the authors propose an electrically responsive self-growing core–shell mushroom-shaped structure with a rigid core and a soft shell, which exhibits excellent adhesion on surfaces with roughness ranging from the nanoscale to the microscale.

## Introduction

Living organisms, such as geckos, spiders, and beetles, have a remarkable climbing ability and can attach to and detach from nearly any surface through the use of van der Waals forces due to the micro/nanostructures on their toe surfaces^1–5^. Inspired by the bioadhesive properties of these organisms, various structured materials have been fabricated with great potential in many areas of robotics, such as climbing robots on vertical walls and grasping or manipulating robots on diverse spatial objects, as well as robots used for production lines and daily life^6–14^. Despite the fact that numerous research works on bioinspired dry adhesives have considerably promoted the development of robotic operation systems, most proposed materials and structures only exhibit high adhesion on smooth surfaces (even exceeding 200 kPa)^15–17^. Indeed, the adhesion strength is reduced by one or two orders of magnitude on rough surfaces and even drops to 0 in the case of rough surfaces with roughness up to dozens of micrometers^18–20^. Achieving a good adhesion on rough surfaces is still a great challenge, which restricts the applicability of robotic operation systems as rough surfaces are more commonly employed in the industry and agriculture fields as well as in people’s daily lives.

In order to reduce the adhesive performance difference between artificial structures and biological ones, various strategies have been proposed for improving the adhesion of artificial structures on rough surfaces. These strategies include lowering the effective elastic modulus via hierarchical structures or soft material-based structures^21–25^, using stiffness-tunable structures for realizing a soft state when contacting the target surface and a stiff state when attaching to it^26–29^, employing separated bilayer structures with a soft top layer and a hard bottom layer for improving the contact area and retaining the structural geometry^30–33^, and utilizing collaborative structures for harnessing the action of the capillary or electrostatic force^34–36^. However, the adaptability of these proposed structures to surfaces with different roughness values (especially for rough surfaces with roughness at dozens or hundreds of micrometers), the reaction time for the attachment/detachment process, and the structural durability are considerably inferior to those of the biological structures. Up to now, no dry adhesives capable of strongly attaching to surfaces with roughness ranging from the nanoscale to the microscale up to hundreds of micrometers while simultaneously exhibiting a fast response and excellent durability have been reported, preventing the development of dry-adhesive-structure-based devices and systems.

The considerable difference between the artificial structures and the biological ones regarding their adhesive performance can be ascribed to their distinct structural configuration and forming mechanisms. (1) Structural configuration. For existing adhesion strategies, artificial structures usually consist of homogeneous materials or separated layers, which exhibit poor adhesion on rough surfaces, as mentioned above. By contrast, biological structures comprise complex geometry and specific stiffness. For instance, the adhesive structures of gecko, spider, beetle, etc., have the tip geometry of their foot with a spatula or mushroom-like morphology^37^. In addition, the structural stiffness varies from a small value at the tip to a large one at the end, as well as the difference even exceeds an order of magnitude^38,39^. The soft part is beneficial for approaching and contacting the target surface, and the rigid part supports the specific morphology and applies a load to hold the contact state. The combined action of different stiffness parts promotes a conformal contact and prevents peeling-off behavior, thereby exhibiting a superior performance, particularly on rough surfaces, especially for rough surfaces with roughness up to dozens or hundreds of micrometers. (2) Forming mechanism. Artificial structures are usually obtained through mechanical fabrication approaches, such as photolithography, etching, molding, and 3D printing. Thus, the geometry and functionality of fabricated artificial structures are limited and are not comparable to those of biological structures. By contrast, biological structures are formed via self-growth in one step rather than a sequence of processes, as in the conventional fabrication strategies. That is, the geometrical shape (spatula or mushroom-like morphology, for instance) and stiffness characteristics (soft–rigid hybrid construction) can be simultaneously generated, which is distinctly different from the conventional methods.

**Fig. 1:**
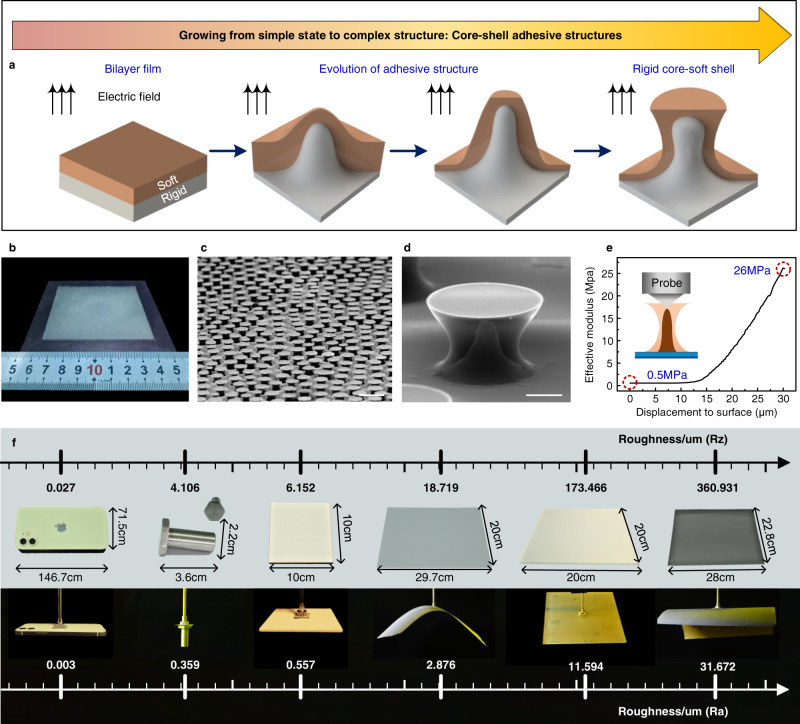
Schematic of the self-growth of adhesive structures with a rigid core and a soft shell. **a** Schematic of the growth process of the proposed core–shell adhesive structure under the action of an external electric field. **b** Photograph of the grown mushroom-shaped structures with a rigid core and a soft shell: the structures have an area of 7 × 7 cm^2^, and TPU and silicon rubber are adopted as the rigid and soft materials, respectively. **c** Distribution of the grown core–shell structures exhibiting a short-range order. The scale bar is 500 μm. **d** Synthetic image of the grown adhesive structure based on separated outer and inner geometries, with the inner geometry obtained by sacrificing the soft materials. The scale bar is 50 μm. **e** Variation in the effective elastic modulus as a function of indentation depth. **f** Demonstration of the grasping ability of the structure on diverse target surfaces, namely a smooth cell phone, a screw nut, an A4 paper, ground glass, and sandpaper.

Here, we propose a core–shell adhesive structure, which is realized via a growth strategy (Fig. 1a), i.e., electrically responsive self-growing core–shell structures, leading to a mushroom-shaped morphology with a rigid core and a soft shell. Initially, the state of the structure consists only of a bilayer film without any specific geometry. When the structure is exposed to an external electric field, the bilayer film grows upward under the action of the electrostatic force. Finally, the top polymer comes into contact with the upper electrode and expands over it due to the electrowetting effect, whereas the bottom polymer continues to grow while being restricted by the top layer, which results in the formation of a mushroom-shaped structure with a rigid core (the bottom layer) and a soft curved shell (the top layer). A schematic of the growth process is provided in Supplementary Fig. 1. The obtained mushroom-shaped geometry demonstrates a superior adhesion compared to other ones (such as flat, spherical, concave, and spatula morphology), because the mushroom-like cap can eliminate the stress singularity at the contacting interface (i.e., homogeneous stress distribution) and stabilize defects at the plate–substrate interface, which has been clearly explained by Gorb and Carbone et al.^40,41^. In addition, the rigid material grows as a mushroom stem, and the soft material grows as a mushroom epidermis surrounding the stem. This structure exhibits a superior adhesive performance on rough surfaces.

Figure 1b–d shows the grown structure with a rigid core and a soft shell under the action of an electric field (different magnification), and Fig. 1e shows the effective elastic modulus of the structure as a function of indentation depth. The core–shell adhesive structure (with an area of 7 × 7 cm^2^) was prepared on an indium tin oxide (ITO)-coated glass substrate via a one-step self-growth approach (thermoplastic urethanes (TPU), and silicon rubber were adopted as the rigid and soft materials, respectively); it would be difficult to realize such a structure via the traditional fabrication methods (Fig. 1b). In general, the mushroom-shaped structures can be obtained via etching^42^, photolithography^43,44^, etc., which, however, is difficult to be used for producing core–shell structures. Similarly, the core–shell structures (compliant coating on more rigid structures) can be generated via molding accompanied by optical alignment operation^45,46^, which is difficult to be used for obtaining mushroom-like geometry at the micro/nano-scale. In contrast, the proposed growth strategy can simultaneously generate the core–shell feature and the mushroom-like morphology in one step. The distribution of the grown structures is characterized by a short-range order due to the combined action of the electric field and the thermal instability (Fig. 1c). Furthermore, the rigid stem is overcovered by the soft material with a specific mushroom-shaped geometry (Fig. 1d), in which the soft-shell material resembles a mushroom cap and the rigid-core material acts as the structural stem. In addition, the variation in the elastic modulus with depth confirms the realization of the rigid core–soft shell structure, i.e., the effective elastic modulus increases from 0.5 to 26 MPa as the testing probe penetrates from the top surface to the bottom domain of the grown structure (Fig. 1e); these are typical values of the intrinsic elastic modulus of soft and rigid materials (Supplementary Fig. 2).

For a smooth target surface, under the influence of preload force, the soft layer conforms to the target surface as the adhesive structure approaches it. Owing to the action of mushroom-like morphology and the rigid core/soft shell configuration, a crack is usually generated in the central section of the interface due to the stress distribution (Supplementary Fig. 3), which is beatifical for enhancing the adhesion on smooth target urfaces^40,41,47^. For a rough target surface, the generation of a crack is also affected by the morphology of target surfaces^40,48^, i.e., the crack may not be generated in the central part. Here, the mushroom-shaped core–shell structure also exhibits superior adhesive performance on rough surfaces with a working mechanism and adhesive performance demonstrated in the following section. Figure 1f demonstrates the good adaptability of the grown core–shell adhesive structure on target surfaces with roughness ranging from the nanoscale to the microscale. To the best of our knowledge, such elevated adaptability has never been achieved using conventional dry adhesion strategies. The targets chosen in this work to illustrate the adhesive capabilities of the proposed structure are commonly used objects in our daily life, i.e., a cell phone (*R*_a_ = 0.003 μm and *R*_z_ = 0.027 μm), a screw nut (*R*_a_ = 0.359 μm and *R*_z_ = 4.106 μm), a ceramic plate (*R*_a_ = 0.557 μm and *R*_z_ = 6.152 μm), an A4 paper (*R*_a_ = 2.876 μm and *R*_z_ = 18.719 μm), a ground glass (*R*_a_ = 11.594 μm and *R*_z_ = 173.466  μm), and sandpaper (*R*_a_ = 31.672 μm and *R*_z_ = 360.931 μm). Here, *R*_a_ is arithmetical mean roughness, and *R*_z_ is ten-point mean roughness.

## Results

### Electrically responsive growth mechanism of the rigid core–soft shell structure

**Fig. 2:**
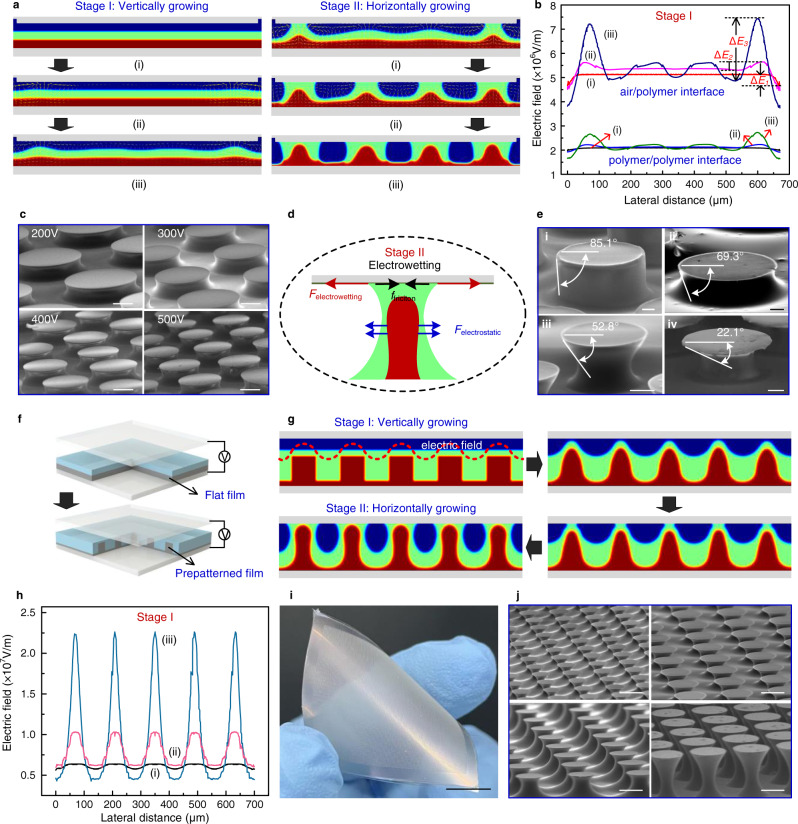
Analysis of the core–shell adhesive structures grown under the action of an electric field. **a** Dynamic evolution of the bilayer polymer film under an external electric field obtained via numerical simulations. Two stages can be distinguished: stage I (vertical-growth stage) and stage II (horizontal-growth stage). Here, the colors of blue, green and crimson represent air, soft polymer, and rigid polymer, respectively. **b** Electric field distribution at the air–polymer, and polymer–polymer interfaces at different snapshots during stage I. **c** Structures grown at different voltages in the range of 200–500 V with intervals of 100 V. The scale bar is 100 μm. **d** Schematic of the mechanical analysis of the bilayer film after contacting the top electrode surface. The colors green and crimson represent soft polymer and rigid polymer, respectively. **e** Core–shell adhesive structures grown at different contact angles, which correspond to diverse voltages. The scale bar is 50 μm. **f** Schematic of the prepatterned bilayer polymer film growth process. **g** Dynamic evolution of prepatterned bilayer polymer film under an external electric field. The colors blue, green, and crimson represent air, soft polymer, and rigid polymer, respectively. **h** Electric field distribution at the air–polymer interface at different snapshots during stage I for the prepattern strategy. **i**, **j** Demonstration that these adhesive structures can be grown with a large area using flexible ITO-coated PET films as conductive substrates (**i**) and different distributions corresponding to distinct prepatterns of the bottom layer (**j**). The scale bars are 1 cm and 100 μm, respectively.

To better understand the growth process of the rigid core–soft shell structure, we propose a numerical model based on a three-phase flow to describe the forming behavior of the bilayer film subjected to an external electric field. This model combines the Gauss equation for representing the electric field, the Navier–Stokes equation for describing the flow field, and the Cahn–Hilliard equation for expressing the behavior of air and the two fluidic materials. The details on the numerical model are provided in Supplementary Note 1. Figure 2a shows the evolution of the bilayer polymer under an external electric field, and the corresponding dynamic motion is shown in Supplementary Movie 1. According to the obtained morphology, the development process can be divided into two stages, namely stage I, in which vertical growth occurs (the growth of the mushroom stem), and stage II, in which horizontal growth occurs (the growth of the mushroom cap). Initially, the flat top layer covers completely the bottom layer. When a voltage is applied, the electrostatic force generated at the air–polymer and polymer–polymer interfaces causes the bilayer film to grow upward the upper electrode by overcoming the viscous force and surface tension; this corresponds to stage I. During this process, the morphology of the bilayer polymeric film changes from flat to structured. Stage II occurs after the top polymer comes into contact with the upper electrode and spreads across the electrode surface due to the electrowetting effect, forming the mushroom cap. At the same time, the rigid polymer continues to grow inside the soft polymer, eventually forming the rigid core–soft shell geometry. Clearly, the structures are first generated near the edge of the electrode and then extend to its center. This phenomenon can be attributed to the variation in the electrostatic force acting on the bilayer film, which is, in turn, dictated by the electric field distribution (Supplementary Fig. 4).

Figure 2b shows the electric field on the fluidic interfaces at stage I. The amplitude of the electric field at the air–polymer interface drops abruptly in the regions near the electrode edges during stage I (i). Owing to the nonuniformity of the electric field near the electrode edges, these edge regions are also the regions in which the film becomes mechanically unstable, and the pillars start to grow first. Once the polymer near the electrode edge starts to flow upward, the film in the vicinity of the template edges tends to flow toward to the electrode edges to supply the polymer. Therefore, the growth of periodic pillars is initialized at the electrode edge and continues toward the central part of the electrode. To describe the evolution of the bilayer film, we define the effective electric intensity as Δ*E* = *E*_max_ − *E*_min_, which corresponds to the effective driving force with *E*_max_ and *E*_min_ denoting the maximum and minimum electric intensity at the interface, respectively. Clearly, Δ*E* becomes larger as the polymer moves upward, i.e., Δ*E*_3_ > Δ*E*_2_ > Δ*E*_1_. Thus, the electric field and the height of the pillars have a positive feedback effect on each other, i.e., a stronger electric field causes the polymer to move upward to a greater extent, and this polymer induces a stronger electric field. It is this mutual positive feedback effect that drives the pillar growth until the bottom surface of the upper electrode is reached. The Δ*E* evolution of the electric field at the polymer–polymer interface is similar. Thus, the polymer–polymer interface also grows from the edge to the center of the electrode, which results in the formation of the core–shell geometry.

The growth behavior is affected by the electric field at the fluidic interface; thus, the periodicity of the grown structure can be controlled by adjusting the voltage (Fig. 2c). Upon increasing the voltage from 200 to 500 V, the periodic length of the structure is decreased by roughly a factor of two due to the increment of the electrostatic force. Notably, only when the relative permittivity of the rigid polymer, *ε*_3_, is larger than that of the soft polymer, *ε*_2_, the anticipated core–shell structure can be generated (Supplementary Fig. 5a). When *ε*_2_ = *ε*_3,_ no electrostatic force is generated at the polymer–polymer interface; thus, the bottom layer flows passively driven by the fluidic behavior of the top layer, which results in the formation of a layered structure rather than an overcovered structure. When *ε*_2_ > *ε*_3_, the electrostatic force generated at the polymer–polymer interface causes the top polymer to grow downward. In this case, despite the grown structure resembling a core–shell structure, the top layer hinders the upward movement of the bottom polymer, causing the inner polymer to reach only a modest height.

After contacting the upper electrode, the top polymer spreads on the electrode surface, driven by the electrowetting effect. This corresponds to stage II (the horizontal-growth stage), in which the electrostatic and electrowetting forces drive the top polymer to overcome the viscous force and friction on the solid surface to move along the electrode surface, finally resulting in the formation of the mushroom cap (Fig. 2d). Simultaneously, the bottom polymer also grows while being restricted by the top polymer, as demonstrated in Fig. 2a. Here, in order to enhance the electrowetting phenomenon, a dielectric layer is coated on the upper electrode surface to introduce the influence of the electrowetting-on-dielectric (EWOD) effect. The electrowetting contact angle (*θ*) at the three-phase boundary can be determined by the Lippmann–Young equation as follows^49^:

1 $$\cos (\theta )=\,\cos ({\theta }_{0})+{F}_{l}/\xi \; {F}_{l}\propto {E}^{2}$$

where *θ*_0_ is the natural contact angle, *ξ* is the surface tension coefficient, and *F*_*l*_ is the electrostatic force acting on the contact line. Further details of this expression are provided in Supplementary Note 1. Consequently, the geometry of the mushroom cap can be modulated by adjusting the contact angle, which is, in turn, controlled by the electrostatic force *F*_*l*_.

Clearly, the variable *F*_*l*_ depends on the electric field, which can be changed by varying the voltage and the distance between the electrode pair. Figure 2e shows the core–shell structures grown at different voltages and electrode distances. At a voltage of 200 V, the contact angle is 85.1° for an electrode distance of 160 μm, which corresponds to a small electric field, whereas the contact angle is 69.3° for an electrode distance of 100 μm, which corresponds to an increased electric field (Fig. 2e-i and ii, respectively). For an electrode distance of 100 μm, the contact angle is 52.8° at a voltage of 1100 V and 22.1° at a voltage of 1500 V (Fig. 2e-iii and iv, respectively). It should be noted that the contact angle shown in Fig. 2c does not change markedly with voltage. In this case, the electrode distance is 70 μm, and the voltage changes from 200 to 500 V; the variation in the electric field is not sufficient to affect the contact angle in a pronounced manner. Thus, the periodicity of the grown structures is more sensitive to the contact angle of polymer on the electrode surface. This offers the possibility to independently modulate the periodicity and cap geometry of the structures to some extent.

In addition to the core–shell structures at the microscale, the proposed growth technique can generate nanoscale structures (Supplementary Fig. 6), with the demonstrated core-shell structures having a characteristic length of about 90 nm from the viewpoint of numerical simulation. In practice, the nanoscale structures can be experimentally realized via precisely controlling the parallelism between electrode pairs and the geometric variables (consisting of polymeric height, air gap, etc.) at the nanoscale, which needs a specific mechanical construction to ensure the rationality of these parameters. Furthermore, the influence of process parameters, such as viscosity, external voltage, air gap, etc., on the electrically growing process is explored, as shown in Supplementary Figs. 7–9, respectively. With an increased viscosity, the viscous force becomes larger, resulting in a larger resistive force to hinder the growing behavior (Supplementary Fig. 7). The growth velocity decreases as the viscosity coefficient rises from 0.02, 0.2 to 2 Pa s, implying that more time is required to generate the core–shell structures. Even when the viscosity coefficient is increased to 20 Pa s, the air–polymer–polymer interfaces remain unchanged (Supplementary Fig. 7b-iv), suggesting that the viscous force is too great to allow the bilayer polymer to move upwards. For the external voltage, it affects the growing process via the electrically driving force (Supplementary Fig. 8). With a small value of 100 V, the driving force is too small to conquer the resistive force, resulting in a fluctuant morphology instead of core–shell structure. With the increment of voltage, the driving force becomes larger and larger, leading to the core–shell structures with an increased dense packing. For the air gap between the upper electrode and the soft polymer, a smaller thickness corresponds to a larger driving force, resulting in core-shell structures with a dense distribution. With the increase of air gap thickness, the driving force gradually decreases, leading to the core-shell structures with dispersive packing and a small growing velocity (Supplementary Fig. 9). Based on the aforementioned discussion, the relationship between the driving force and resistive force determines the growing process, i.e., a large driving force corresponds to core-shell structures with dense packing and a fast-growing, and a large resistive force leads to the structures with dispersive packing and a slow growing. Thus, the growing process can be modulated by adjusting the relation of driving force and resistive force, which is further influenced by process parameters (such as viscosity coefficient, external voltage, air gap).

It should be noted that the short-range order is a critical characteristic of the grown core–shell structures for the flat bilayer film due to no constraint on the electric field. Thus, the diameter and packing of core-shell structures may not be regular (Fig. 1d and Supplementary Fig. 10). Here, we propose a different strategy, which we call the prepattern strategy, i.e., a prepatterned bottom layer instead of the flat bottom layer, to provide a modulated electric field to control the polymer evolution (Fig. 2f). The schematic of the forming process is shown in Supplementary Fig. 11. In this case, the electric field aligns with the prepattern and drives the bilayer polymer to move upward as the distribution of the prepatterns on the bottom layer. Figure 2g illustrates the evolution of the prepatterned bilayer polymer under an electric field, and the corresponding dynamic motion is shown in Supplementary Movie 2. The dynamic evolution is similar to that of the flat structure except for the fact that the pillars grow simultaneously rather than sequentially from the edge to the center of the electrode. For demonstrating the superiority of the prepattern strategy on the regularity of core-shell structures, we explored the formation of prepatterned bilayer film with different voltages (Supplementary Fig. 12) as compared with the flat case (Supplementary Fig. 8b). Clearly, the core-shell structures cannot be generated with a low voltage for both prepatterned and flat configurations. Furthermore, despite the anticipated structures are both obtained for increasing the voltage, the regularity of the flat case is far less than that of prepatterned one, i.e., the prepatterned film-generated structure precisely corresponds to the initial structure, and the flat bilayer-generated structures exhibit slight difference on diameter and periodicity. This phenomenon can be attributed to the spatially modulated electric field incurred by prepatterns of the bottom film.

The evolution of the electric field at the air–polymer, and polymer–polymer interfaces also exhibits a positive-feedback relationship with the pillar height until the pillars touch the upper electrode (Fig. 2h and Supplementary Fig. 13). The driving force is the electrostatic force, and the main resistive force is the surface tension in the vertical-growth stage; thus, the fluidic behavior of the prepatterned bilayer film can also be discussed in terms of a force analysis (the details of this analysis are provided in Supplementary Note 2). Regarding the relative permittivity of the bilayer film, the anticipated core–shell structures can be obtained only if *ε*_2_ < *ε*_3_ (Supplementary Fig. 5b), which is similar to the phenomenon of the flat bilayer film. When *ε*_2_ = *ε*_3_, no electrostatic force is generated at the polymer–polymer interface; thus, the bottom layer flows passively, driven by the fluidic behavior of the top layer, preventing the formation of the overcovered structure. When *ε*_2_ > *ε*_3_, the electrostatic force generated at the polymer–polymer interface causes the top polymer to grow downward, which results in the formation of a layered structure rather than an overcovered structure.

Based on the tunability of the vertical-growth stage for the mushroom stem and the horizontal-growth stage for the mushroom cap, the geometry of the rigid core–soft shell structure can be modulated by adjusting the process parameters. Here, a rigid core–soft shell adhesive structure with an area of 5 × 5 cm^2^ was prepared on a flexible ITO-coated PET substrate via the self-growth approach (Fig. 2i), with the distribution of the grown structure corresponding to that of the initial bottom structure. The detailed parameters, including the height, diameter, and separating distance of core-shell structures, can be determined by varying the initial pattern, which results in the formation of different geometrical structures exhibiting diverse adhesive performances (Fig. 2j). This highlights the flexibility of the self-growth technique for adjusting the adhesive strength according to the requirements of practical applications.

### Adhesion enhancement mechanism of the grown core–shell structures

**Fig. 3:**
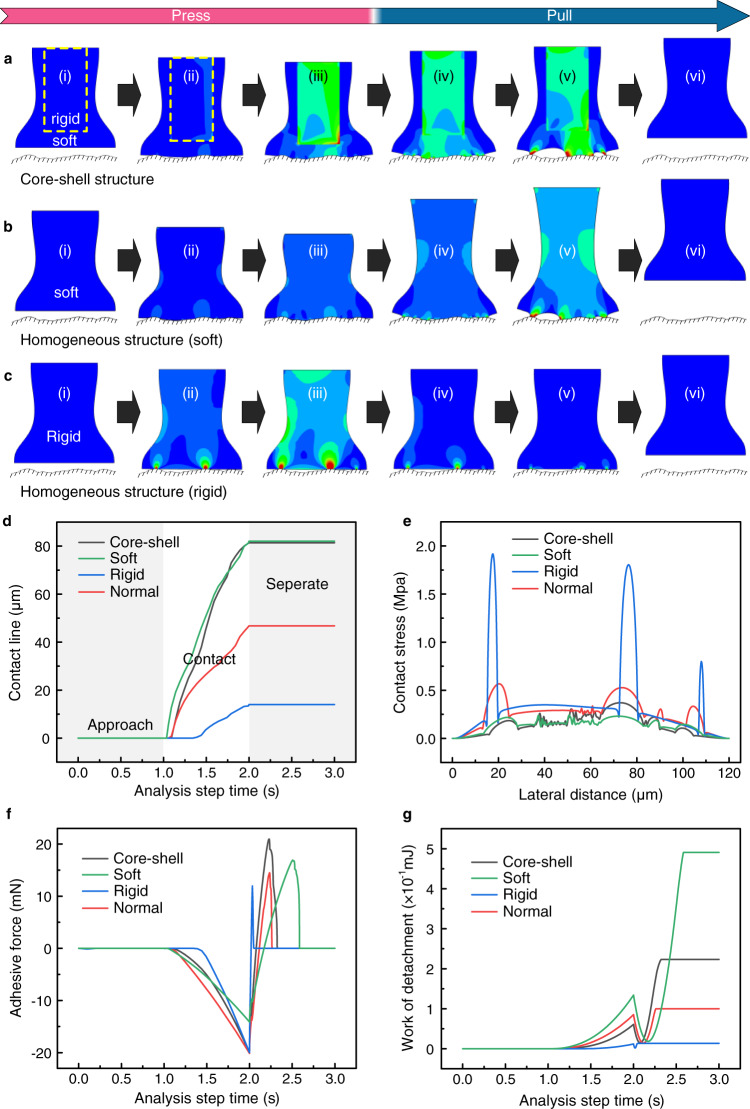
Adhesion enhancement mechanism of the grown core–shell structures on rough surfaces. **a**–**c** Dynamic behavior of the core–shell structure (**a**), soft structure (**b**), and rigid structure (**c**) on rough surfaces when (i) approaching, (ii and iii) contacting, and (iv–vi) separating from the rough surface; cloud atlas representing the internal stress. **d** Evolution of the contact line for different adhesive structure as a function of the process time. **e** Stress at the interface between the adhesive structure and the rough surface. **f** Adhesive force as a function of the processing time for different adhesive structures. **g** Work of attachment/detachment as a function of the processing time for different adhesive structures. Here, core–shell, soft, rigid, and normal represent a mushroom-shaped structure with a rigid core and a soft shell, mushroom-shaped structure with soft material, mushroom-shaped structure with rigid material, and mushroom-shaped structure with common elastic material, respectively.

To investigate the adhesion enhancement mechanism of the mushroom-shaped core–shell structure on rough surfaces, we implement a numerical model based on the interfacial cohesive zone theory to analyze the contact–separation process for four scenarios, consisting of mushroom-shaped structure with a rigid core and a soft shell, mushroom-shape structure with soft material (corresponding to the soft material of the core-shell structure), mushroom-shape structure with a rigid material (corresponding to the rigid material of the core-shell structure), and mushroom-shaped structure with common elastic material, which are abbreviated as core–shell structure, soft structure, rigid structure, and normal structure, respectively. The details are provided in Supplementary Note 3. Here, due to the collapse of the soft pillars, the soft material from the soft part of core–shell structure is difficult to be experimentally fabricated as a mushroom-like structure. Thus, we additionally adopted the common elastomer material (polydimethylsiloxane, polyurethane acrylate, etc.) as the comparison. Figure 3a–c and Supplementary Fig. 14 illustrate the dynamic behavior of the four abovementioned adhesive structures contacting a rough surface and separating from it, with the cloud atlas representing the stress distribution. The corresponding dynamic evolutions are presented in Supplementary Movies 3–6, respectively. Here, the morphology of the target rough surface was imported from ground glass via laser scanning confocal microscopy.

During the pressing/attachment process, the internal stress within four adhesive structures increases from 0 to a large value as the indentation depth increases, whereas the stress distributions in the core–shell structure and soft one are more uniform than those in the rigid and normal structures (Figs. 3a-ii, 3b-ii, 3c-ii and Supplementary Fig. 14-ii). This phenomenon indicates that a larger contact area can be obtained for the soft contact. As can be inferred by comparing Fig. 3a-iii, 3b-iii, 3c-iii and Supplementary Fig. 14-iii, a finer contact status occurs in the case of the core–shell structure and soft one, i.e., soft contact cases. The evolution of the contact line representing the four adhesive structures is displayed in Fig. 3d, in which the approach, contact, and separation processes can be distinguished with an analysis step time of 1 s interval. In order to highlight the capability of conformal contact for different adhesive structures, the contact line is plotted as a constant value (corresponding to the maximum contact line) at the separation process. The contact line remains at 0 at the time of 0–1 s since there is no contact between the adhesive structure and the rough surface. Starting from 1 s, the contact line becomes increasingly large with increasing time up to 2 s, at which point the adhesive structure starts to detach from the rough surface, which corresponds to the snapshot with the maximum contact area. Owing to the action of the soft layer, the contact line of the core–shell and soft structure is nearly two times as large as that of normal structure and six times for rigid structure. Here, the adhesive structures were pushed to contact the rough surface and separate from it by a set distance, and the preload force was calculated in this process. The variation in displacement as a function of time is shown in Supplementary Fig. 15. In addition, the preloads acting on the core-shell, rigid and normal structures were set as identical to evaluate the influence of soft parts on the contact status. For the soft structure, a small preload force would cause an obvious deformation due to the low modulus. Thus, we adopted conformal contact as the criterion to set the preload on the soft structure. The contact status can also be evaluated by interfacial stress at the contacting surface (Fig. 3e). Obviously, the differences in stress for the rigid structure and normal one are considerably larger than those for the core–shell and soft structures, which also indicates that the soft part is easy for the conformal contact on the rough surface. Additionally, the difference for soft structure is slightly less than that of core-shell structure, implying that soft structure may be able to attain finer contact status on a rougher surface.

During the pulling/detachment process, the core–shell structure and soft one still have a larger contact area than the rigid and normal structures at the moment of the detachment starting to occur (Fig. 3a–iv, 3b-iv, 3c-iv and Supplementary Fig. 14-iv). Notably, despite having introduced the mushroom-shaped geometry into four adhesive structures, the initial cracks are not generated in the central region of the mushroom-shaped cap. The generation of cracks depends to some extent on the morphology of the target surface, as shown in Fig. 3a-v, 3b-v, 3c-v and Supplementary Fig. 14-v, which differs from the detachment behavior of the mushroom-shaped structure from smooth surfaces. Finally, after being completely disengaged from the rough surfaces, the adhesive samples recover their original shape due to the relaxation of the elastic energy accumulated during the pressing process (Fig. 3a-vi, 3b-vi, c-vi and Supplementary Fig. 14-vi). Although the difference in interfacial stress corresponding to the soft structure is smaller than that of the core-shell (Fig. 3e), the adhesive force of core–shell structure is inversely larger than that of the soft structure (Fig. 3f). Thus, it can be inferred that the contact area on the target surface is a critical parameter to influence the adhesion, but is not the only factor. The adhesive force for different configurations is listed from a large value to a small as a sequence of core-shell, soft, normal, and rigid. Compared with rigid and normal structures, the superior of core-shell structure on the adhesion can be attributed to the effective contact area in the attachment process, which has been discussed in Fig. 3d, e. Compared with the soft structure that almost has the same contact area, the enhancement of adhesion for core-shell structure may be attributed to the action of structural stiffness^50,51^. For a high stiffness, the adhesive structure tends to be separated from the target surface as a whole part, which is advantageous for preventing peeling-off behavior. In contrast, with low stiffness, the peeling-off behavior can easily occur starting from the contact point with high interfacial stress. The comparison of the detachment process of core–shell structure and soft structure can be observed via the dynamic evolutions (Supplementary Movies 3 and 4). Furthermore, in order to explore the action of structural stiffness on the adhesive force, we performed the numerical simulations on core-shell structures that have core pillars with different heights (Supplementary Fig. 16a). With the increment of the core pillar height, the adhesive force becomes larger, demonstrating the positive effect of high stiffness on the adhesion. However, if the adhesive structure becomes completely rigid, the adhesive force would be smaller than the soft structure due to a small contact area (Fig. 3d). Consequently, for core-shell structures, an effective approach to enhance the adhesive force is to increase the rigid core pillar height without dramatically decreasing the contact area between adhesive structures and rough surfaces.

Figure 3g demonstrates the work of attachment and detachment of adhesive structures on rough surfaces. Owing to the state transition from attachment to detachment, there is an obvious turning point in the curve of the external work, i.e., the value of work sequentially becomes from small to large, from large to small, and then to larger. Despite the adhesive force of core–shell structure is greater than that of the soft structure, the work of detachment for core–shell structure is smaller than that of the soft structure, which can be attributed to the larger elastic deformation of the soft structure (Fig. 3a-v and 3b-v). The influence of structural stiffness on the work of detachment is demonstrated in Supplementary Fig. 16b, in which the variation of stiffness is also achieved via controlling the height of the rigid core pillar. With the decrease of the core pillar height, the work of detachment becomes larger, i.e., a low stiffness is beneficial for increasing the work of detachment. Based on the comparison of adhesive force and work of detachment for core-shell structure and soft structure, it can be inferred that the core-shell structure with mushroom-shaped geometry exhibits superior adhesive force because of the high stiffness, and the soft structure with mushroom-shaped geometry appears better work of detachment owing to the low stiffness.

### Adhesive performance of the grown core–shell structures on rough surfaces

**Fig. 4:**
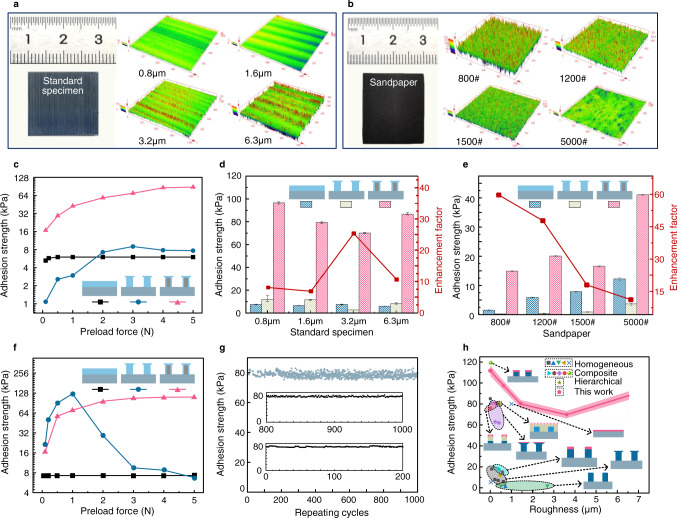
Adhesion of the grown core–shell structures on diverse rough surfaces. **a**, **b** Target rough surfaces used in the experiments, including standard specimens and sandpaper, respectively. **c** Variation in the adhesion strength as a function of the preload force for the tested samples, namely the core–shell structure, homogeneous structure, and soft polymer film. **d**, **e** Adhesion strength of the three samples on standard specimens and sandpaper, respectively. Error bars are defined as the difference between the limiting value of adhesion strength and the average value of adhesion strength. **f** Variation in the adhesive force on smooth glass as a function of the preload force. **g** Test cycles of the adhesion performance of the core–shell structure on the rough surfaces of standard specimens. **h** Comparison of the results obtained in this study with those reported by previous works on target surfaces with roughness ranging from the nanoscale to the microscale.

To evaluate the adhesive performance of the prepared rigid core–soft shell structures on rough surfaces, we tested three different samples, namely a mushroom-shaped structure with a rigid core and a soft shell, a mushroom-shape structure with a homogeneous material (corresponding to that of the normal structure shown in Fig. 3), and a flat film with a soft material. Here, a flat film based on soft material rather than soft pillars was adopted as the testing structure due to the collapse of the soft pillars. In detail, standard specimens with nominal roughness of 0.8 μm (*R*_a_ = 0.627 μm, *R*_z_ = 4.079 μm), 1.6 μm (*R*_a_ = 1.43 μm, *R*_z_ = 16.961 μm), 3.2 μm (*R*_a_ = 3.593 μm, *R*_z_ = 28.505 μm), and 6.3 μm (*R*_a_ = 6.549 μm, *R*_z_ = 4.079 μm) (Fig. 4a) as well as sandpaper with 800# (*R*_a_ = 23.6 μm, *R*_z_ = 238.948 μm), 1200# (*R*_a_ = 9.6 μm, *R*_z_ = 113.726 μm), 1500# (*R*_a_ = 4.5 μm, *R*_z_ = 43.446 μm), and 5000# (*R*_a_ = 3.5 μm, *R*_z_ = 21.198 μm) (Fig. 4b) were selected as the target rough surfaces. In addition, the power special density (PSD) of the standard specimens and sandpaper is demonstrated in Supplementary Fig. 17a, c, respectively, as well as the detailed morphology of rough surfaces obtained by laser scanning confocal microscope (Supplementary Fig. 17b, d). It can be found that the wavelength of rough surfaces is not much larger than the lateral size of the core-shell structure. Notably, the *R*_z_ roughness value of the 800# sandpaper is as large as 240 μm; to the best of our knowledge, such a rough surface with roughness at hundreds of micrometers has never been adhered to by conventional dry adhesive structures. Figure 4c demonstrates the adhesive force of the three different samples on the 6.3-μm standard specimen with increasing preload. It can be seen that for all samples, the adhesive force first increases and then becomes constant with increasing preload. For a small preload, the core–shell structure exhibits the greatest adhesion, followed by those of the soft polymer film and the homogeneous structure, which indicates that the presence of the soft material is beneficial for increasing the contact area. For a high preload, the core–shell structure still exhibits the greatest adhesion, followed by those of the homogeneous structure and the soft polymer film, which reflects the influence of the mushroom geometry on the adhesive performance. Importantly, the smallest adhesive force of the core–shell structure is larger than the greatest adhesive force of the homogeneous structure and the soft polymer film by almost a factor of two, regardless of the preload value. For the same preload, the enhancement factor can reach one order of magnitude. In the following discussion, the saturation value of the adhesive strength for all samples is taken as the adhesive strength.

Figure 4d illustrates the adhesive performance of the three different samples on the standard specimens; the adhesive strength of the core–shell structure on the 0.8-, 1.6-, 3.2-, and 6.3-μm specimens is 96.67, 79.42, 70.17, and 86.67 kPa, respectively, which is about one order of magnitude higher than those of the homogeneous structure and the soft polymer film, reaching almost the adhesion strength of the gecko’s foot^1,52^. As the roughness increases, the enhancement of the adhesive strength becomes more pronounced, increasing from a factor of eight for the 0.8-μm specimen to a factor of 10.6 for the 6.3-μm specimen; this further demonstrates the adaptability of the core–shell structure. Furthermore, the core–shell adhesive structures exhibit an enhancement of the adhesive strength also for surfaces with a higher roughness, such as sandpaper (Fig. 4e). For the 800# sandpaper with *R*_a_ = 23.6 μm and *R*_z_ = 238.9 μm, the adhesive strength of the core–shell structure is 14.92 kPa, whereas it is only 1.5 kPa for the soft polymer film and 0.25 kPa for the homogeneous structure, corresponding to increments of roughly a factor of 10 and a factor of 60, respectively. Surfaces with *R*_z_ roughness values amounting to hundreds of micrometers have rarely been studied and seldom selected for dry adhesion tests. The increased adhesion of the core–shell structure on 1200#, 1500#, and 5000# sandpaper is also noticeable when compared with those of the homogenous structure and the soft polymer film.

Owing to the supporting action of the rigid part inside the mushroom-shaped core–shell structure, this structure can retain a high adhesive force even for a significant preload force, thereby avoiding bending or buckling, as shown in Fig. 4f (a smooth glass is used as the testing surface). For the conventional mushroom-shaped homogeneous structures, the adhesive force is considerably reduced as the preload force exceeds a threshold value, which leads to an undesired structural deformation (such as bending or buckling)^53,54^. The capability of the core–shell structures to withstand bending and buckling deformations makes them promising candidates for use in high-impact scenarios, which is not the case for the conventional dry adhesion structures. Figure 4g illustrates the repeatability of the grown core–shell structures, with their adhesive force remaining unchanged after 1000 cycles. This indicates that the bonding between the rigid and soft materials is sufficiently strong to connect the outer and inner layers. This superior bonding behavior may be due to two factors: (1) the overcovered structure provides an increased contact interface between the rigid and soft materials, and (2) the rigid and soft materials are formed simultaneously in one step through electrical growth rather than a sequence of processes as in the conventional fabrication strategies.

To illustrate the superior adhesion of the grown core–shell structures on rough surfaces, we compare the adhesive strength of the core–shell structures with those of other typical adhesive structures, such as homogenous structures^18,19,55–58^, composite structures^28,30,32,45,59^, and hierarchical structures^60^ (Fig. 4h). Conventional adhesive structures have shown to have limited applicability regarding the roughness of the target surface, i.e., the roughness of the target surface is usually smaller than 1 μm, and the adhesive strength is low. The core–shell structures, on the other hand, can be employed on rough surfaces with roughness ranging from the nanoscale to the microscale and exhibit an increased adhesive force. This comparison demonstrates the good performance of the grown adhesive structures in terms of the range of roughness to which the structures can adhere and the adhesive strength, especially for rough surfaces with roughness up to dozens or hundreds of micrometers.

## Discussion

In summary, we proposed a core–shell adhesive structure generated via the self-growth strategy, i.e., the formation of a complex morphology from a simple one. The obtained mushroom-like morphology is beneficial for equal load sharing across the interface; the soft material promotes a conformal contact, while the rigid material permits the mushroom geometry to be retained and prevents the occurrence the peeling-off behavior. In the growth process, an electric field is applied to generate an electrostatic force, which drives the bilayer film to first grow upward on the upper electrode (generating the mushroom stem) and then grow horizontally on the electrode surface (generating the mushroom cap). In order to improve the controllability of the grown adhesive structures, the prepatterned bilayer film is introduced to replace the flat bilayer film to provide an initially modulated electric field, which drives the polymer to move upward as the distribution of the prepattern on the bottom layer. The morphology of the rigid core–soft shell structure, which is defined by the diameter and height of the mushroom stem, the diameter of the mushroom cap, and the distance between the microstructures, can be modified by adjusting the process parameters, such as the external voltage, air gap, polymer thickness, and dielectric permittivity. This indicates that the adhesive performance of the rigid core–soft shell structure can be modulated by the growth parameters, which cannot be easily achieved using conventional fabrication methods.

A composite post with a stiff core and compliant shell has been proposed for enhanced and tunable adhesion on smooth surfaces^45,46^, demonstrating an excellent adhesive performance coupling high adhesion and effective switchability of attachment/detachment on smooth surfaces. At first glance, the configuration of stiff and compliant parts seems similar to our proposed structure, however, there are some notable differences in the aspects of the designed strategy and fabricating method. (1) Designed strategy. The composite post is designed for a high adhesion on smooth surfaces by adjusting the peak stress on the adhered interface from the edge of the structure to the center, just like the action of mushroom-like geometry on stress distribution. The research is concentrated on the detachment behavior with little attention on the attachment due to the phenomenon of no hindrance on the conformal contact as approaching smooth surfaces, neglecting the impact of surface roughness on the contact status. Hence, the flat cap, instead of mushroom-like geometry, is designed as the morphology of soft materials. Furthermore, the optimized geometry of composite posts may not be suitable for rough surfaces. For instance, a thinner shell thickness is convenient for enhancing adhesion on smooth surfaces, however, it would decrease the effective contact area on rough surfaces; if a thicker soft layer is adopted, the modulation on the peak stress becomes weak, maybe still leading to poor adhesion. Based on the fact that the surface roughness also affects the stress distribution along the adhered interface^40,41,47,48^, our proposed structure (mushroom−shaped core−shell structures) is designed from the viewpoint of increasing contact area in the attachment process and preventing peeling-off behavior in the detachment process. In the attachment process, the soft material is used for conformal contact; in the detachment process, the mushroom-like morphology is beneficial to weaken the stress singularity along the interface and the rigid material is adopted for increasing the structural stiffness, which can both prevent the occurrence of the peeling-off behavior. The effective approach of our proposed structures to enhance the adhesive force on rough surfaces is to increase the rigid core pillar height without dramatically decreasing the effective contact area, which is different from that of the conventional composite post (i.e., the action of mushroom-like morphology can guarantee the peak stress near the center even with a thicker soft shell). (2) Fabricating method. The composite structure with a flat cap is fabricated via multiple molding accompanied by optical alignment operation, which can be attributed to a traditional mechanical fabrication approach. For some structures with a specific morphology at the micro/nano-scale (such as our proposed structure), it is difficult to realize the designed structure via traditional methods. In contrast, our proposed rigid core–soft shell structure with mushroom-shaped morphology is formed in one step through an electrically growth approach, i.e., the geometrical shape and stiffness characteristics can be simultaneously generated via the self-growing approach, which is distinctly different from the traditional methods and can be regarded as the most obvious difference between our work and the previous work.

The grown rigid core–soft shell structures have superior adhesive performance on various surfaces, such as smooth glass, standard specimens, sandpaper, A4 paper, and ground glass, with a maximum adhesive strength of up to 80–90 kPa, which is around 10 times higher than those of homogeneous and soft structures and is almost comparable to that of the gecko’s foot. Furthermore, the grown rigid core–soft shell structures can be utilized on surfaces with roughness ranging from the nanoscale to the microscale up to dozens of micrometers. In particular, for an 800# sandpaper with *R*_a_ = 23.6 μm and *R*_z_ = 238.9 μm, the adhesive strength can be as high as 14.92 kPa, which has never been reported for conventional dry adhesive structures. Furthermore, the adhesive force of the rigid core–soft shell structure remains unchanged after 1000 cycles, demonstrating a remarkable reproducibility, which is critical in robotic applications. The proposed adhesion strategy can promote the application of dry adhesives to a wide range of material surfaces, from smooth surfaces to rough surfaces with multiscale roughness (especially for rough surfaces with roughness up to dozens or hundreds of micrometers), opening an avenue for the development of dry adhesives.

## Methods

### Materials

Unless stated otherwise, solvents and chemicals were obtained commercially and used without further purification. The soft polymer was obtained by mixing silicone gel (80 wt%), and polydimethylsiloxane (PDMS) (20 wt%), in which silicone gel (9400) was obtained from Hongyejie Co., Ltd. (China), and PDMS (Dow Corning Sylgard 184) obtained from Dow Corning Inc. (USA). Thermoplastic polyurethanes (TPU) power (255) was obtained from Bayer (China) Limited, acting as the rigid polymer. Amorphous fluoroplastics solution (AFs) (6 wt%, AF1601) was obtained from the Chemours Company (USA), which acts as the dielectric layer coated on the bottom surface of the upper electrode. Standard specimens of the metal rough surfaces were obtained from Weifang Huaguang Measuring Tools Co., Ltd. (China). Sandpapers (Warrior sandpaper) were obtained from Suzhou Suboli Abrasives Co., Ltd. (China). ITO glass and ITO-coated PET film were obtained from Luoyang Guluo Glass Co., Ltd. (China), which are both acting as electrodes.

### The electrically responsive growth process

The TPU powders were placed on the surface of the ITO-coated glass or ITO-coated PET film and heated to 85 °C for melting. Next, a flat PDMS mold was pressed to the melted polymer for obtaining a flat TPU film that acts as a rigid polymer. The soft polymer was then spin-coated on the TPU film for obtaining the initial bilayer film. Subsequently, PI film acting as a dielectric spacer was placed between the electrode pairs to form the sandwich configuration, composed of upper electrode/air gap/soft polymer/TPU/lower electrode, for the subsequent electrical growth process. Here, a Teflon film with a thickness of roughly 100 nm was coated to the bottom surface of the upper electrode for introducing the electrowetting effect and also beneficial for removing the upper electrode after the growth process. The sandwich configuration was then placed into the oven (85 °C) with an applied DC voltage for several minutes, during which the TPU could flow again under a melting state. After the bilayer film grew to core–shell structures, the temperature was increased to 90 °C for curing the soft polymer. Subsequently, the temperature of the bilayer film was decreased to room temperature for curing the TPU. In the curing process, the voltage was continuously exerted on the electrode pairs for maintaining the polymeric morphology. After removing the upper electrode, the mushroom–shaped structures with rigid core–soft shells were obtained. For the prepatterned bilayer film, the process is similar to that of flat bilayer film except for obtaining the prepatterns on the bottom layer first. In detail, the TPU powders were placed on the surface of the ITO-coated glass or ITO-coated PET film and were heated to 85 °C for melting. Then, a structured PDMS mold was pressed to the melted polymer for obtaining microstructures that acts as the prepattern. The subsequent procedure is identical to that of flat bilayer film.

### Structures characterization

The microstructure of the adhesive material was observed by scanning electron microscopy (SU8010, Hitachi, Japan). The adhesion force of the material was characterized by a computer servo pull–pressure test machine (PT–1176, Baoda, China). The roughness of testing surfaces was obtained by laser scanning confocal microscope (OLS4000, Olympus, Japan). For a synthetic image of the grown core–shell structure, the SEM of the outer geometry was first obtained; then, the soft materials were removed by ultrasonic cleaning using acetone to obtain the SEM of the rigid part; finally, the independent outer and inner geometry were assembled together. The spatial variation of the local elastic modulus along the grown core–shell structure was quantitatively evaluated by quasi-static nanoindentation tests. Displacement-controlled nanoindentation tests were performed on the core–shell structure from tip to base by Nano Indenter (G200, Agilent, USA). The tests were implemented at a maximum indentation depth of 30 μm and a loading/unloading rate of 100 nm/s using a Berkovich tip (tip radius of ∼100 nm). The tensile elastic modulus of soft materials and rigid materials was tested by computer servo pull–pressure test machine (PT–1176, Baoda, China).

### Adhesion measurement and characterization

The adhesion of grown core–shell structures was measured by Load–Pull mode; that is, the probe is first contacted with the sample to generate a certain contact area, and then the reverse movement is performed until complete separation. The maximum tensile force generated before separation was defined as the maximum adhesion force. The probe is made of different surfaces (standard specimen, sandpaper, and glass), with a testing area of 2 mm × 2 mm. The core–shell structures were attached on the base and adjusted to be parallel to the probe surface. The testing surface was moved down at a speed of 1 mm/min to contact with the sample and reached a defined preload for maintaining 5 s, then moved up until the testing surface was completely separated from the core–shell structures. The adhesive force can be deduced via the time–force curve. For the comparison of homogeneous structures and soft polymer film on target surfaces, the testing process was identical to that of core–shell structures.

## Supplementary information

Supplementary Information

Description of Additional Supplementary Files

Supplementary Movie 1

Supplementary Movie 2

Supplementary Movie 3

Supplementary Movie 4

Supplementary Movie 5

Supplementary Movie 6

## Data Availability

All data needed to evaluate the conclusions in this study are present within the article and Supplementary Information. Source data for the main figure and Supplementary Information are provided in this paper. Source data are provided in this paper.

## Source data

Source Data
