# Supplementary material for: Core–shell dry adhesives for rough surfaces via electrically responsive self-growing strategy
Source: Nat Commun. 2022 Dec 10;13:7659. doi: 10.1038/s41467-022-35436-6 (PMC9741600; doi:10.1038/s41467-022-35436-6)
Supplement: Supplementary file 1 — Supplementary Information [file 41467_2022_35436_MOESM1_ESM.pdf]

Supplementary Information for

Core–shell dry adhesives for rough surfaces via electrically responsive self-growing strategy

Hongmiao Tian[1], Duorui Wang[1,2], Yahui Zhang[1], Yuanze Jiang[1], Tianci Liu[1], Xiangming Li[1,2], Chunhui Wang[1], Xiaoliang Chen[1,2] and Jinyou Shao[1,2]*

[1]Micro-and Nano-technology Research Center, State Key Laboratory for Manufacturing Systems Engineering, Xi'an Jiaotong University, Xi'an, Shaanxi 710049, China.

[2]Frontier Institute of Science and Technology (FIST), Xi'an Jiaotong University, Xi'an, Shaanxi 710049, China.

*Corresponding author. Email: jyshao@mail.xjtu.edu.cn

This file contains following information:

Supplementary Notes

Supplementary Note 1: Numerical analysis of growth process for bilayer polymer under an electric field

Supplementary Note 2: Analysis of effective driving force at the air–polymer interface and polymer–polymer interface versus the polymeric height for prepatterned case

Supplementary Note 3: Numerical analysis of grown core–shell structures contacting to and separating from rough surface

Supplementary Figures

Supplementary Figure 1: Schematic of growth process of the mushroom–shaped structures with rigid core–soft shell.

Supplementary Figure 2: Stress–strain curves of polymer used in the experiments.

Supplementary Figure 3: Demonstration of the crack generated in the central section of the interface between the core-shell structure and the smooth surface.

Supplementary Figure 4: Distribution of electric field and electrostatic force on the air–polymer interface and polymer–polymer interface at the initial growing stage.

Supplementary Figure 5: Polymeric deformation of bilayer film with different relative permittivity.

Supplementary Figure 6: Grown core–shell structures with a characteristic length of roughly 90 nm from the viewpoint of numerical simulation.

Supplementary Figure 7: Grown core–shell structures with different viscosity coefficient.

Supplementary Figure 8: Grown core–shell structures with different external voltage.

Supplementary Figure 9: Grown core–shell structures with different air gap between the upper electrode and the soft polymer.

Supplementary Figure 10: Grown core–shell structures from the top view.

Supplementary Figure 11: Schematic of growth process of the mushroom–shaped structures with rigid core–soft shell for prepatterned case.

Supplementary Figure 12: Grown core–shell structures under different external voltage for prepatterned case.

Supplementary Figure 13: Electric field distribution on the polymer–polymer interface at different snapshot of stage I for the prepatterned case.

Supplementary Figure 14: Dynamic behavior of the normal structure on rough surfaces.

Supplementary Figure 15: Variation of displacement versus analysis step time in the numerical simulations.

Supplementary Figure 16: Influence of structural stiffness on the adhesion with the varied stiffness achieved by adjusting the height of rigid core pillar.

Supplementary Figure 17: Characteristics of rough surfaces used for testing the adhesive force of different adhesive structures.

Supplementary Figure 18: Schematic of initial geometry for flat bilayer film and prepatterned bilayer film under an exerted electric field.

Supplementary Figure 19: Variation of effective driving force at the air–polymer interface and polymer–polymer interface versus the polymeric height.

Supplementary Figure 20: Schematic of core–shell structure and meshed core–shell structure.

Supplementary Figure 21: Sketch of traction-separation response for cohesive behavior of adhesive structures in the numerical simulations.

Supplementary Tables

Supplementary Table 1: Parameters used in numerical simulation of growth process

Supplementary Table 2: Parameters used in numerical comparisons of growth process

Supplementary Table 3: Hyperelastic models and material properties for mechanical analysis of core-shell structures

Supplementary Note 1. Numerical analysis of growth process for bilayer polymer under an electric field

The Cahn–Hilliard model, which assumes that the interface thickness between two phases in a system is small but greater than the actual physical one, was used to represent the three immiscible components, namely air, soft material, and hard material. A smooth function φ_i, called as the order parameter, is used to geometrically define one phase. This function is equal to 1 in the phase *i* and 0 outside of it, and it varies continuously in the interfaces between the phase *i* and the others. From a mathematical point of view, this approach may be understood as a regularization of sharp interface models with small interfacial thickness. In detail, three order parameters, φ_1, φ_2, and φ_3 for air, soft polymer and rigid polymer, respectively, each representing the volume concentration of one component, are used to describe a ternary system with a definition of $\varphi_1+\varphi_2+\varphi_3=1$.

The three phase of air and two fluidic polymers can be described via ternary Cahn–Hilliard equation, as follows[1]:

$$\begin{cases} \dfrac{\partial \varphi_i}{\partial t} = \nabla \cdot (\dfrac{M_0}{\Sigma_i} \nabla G_i) \\ G_i = \dfrac{4\Sigma_T}{\gamma} \sum_{j \neq i} \left(\dfrac{1}{\Sigma_j} \left(\partial_i f(\varphi) - \partial_j f(\varphi) \right) \right) - \dfrac{3}{4} \gamma \Sigma_i \Delta \varphi_i \end{cases} \tag{1}$$

wherein φ is the phase function (i.e., order parameter), M_0 is interface mobility (also depended on φ), G is chemical potential, γ is the interfacial thickness, Σ_T is defined by $\frac{3}{\Sigma_T} = \frac{1}{\Sigma_1} + \frac{1}{\Sigma_2} + \frac{1}{\Sigma_3}$ and $\forall i \in \{1,\ 2,\ 3\}$ denotes the fluid of air, soft polymer and rigid polymer, respectively.

The contact angle on the three–phase boundary can also be obtained via order parameter of φ_i, with the expression of

$$\begin{cases} \boldsymbol{n}\cdot\frac{M_0}{\Sigma_i}\nabla G_{\ i}=0 \\ \boldsymbol{n}\cdot\frac{3}{4}\gamma\,\Sigma_i\,\nabla\varphi_i=\frac{3}{4}\gamma\,\Sigma_i\,|\nabla\varphi_i|\cos(\theta_i) \end{cases} \tag{2}$$

with $\boldsymbol{n}$ denoting unit normal vector and θ_i representing the contact angle on the solid wall.

The Cahn–Hilliard system is then coupled with the Navier–Stokes equations to complete the modelling of three–phase incompressible flows. As a result, a convective term is added in the order parameters evolution equations and a capillary force of F_{ca} as the momentum balance, by[2]

$$F_{ca}=G_1\nabla\varphi_1+G_2\nabla\varphi_2+G_2\nabla\varphi_2 \tag{3}$$

This force is a volume approximation of the surface tension force, i.e., in this model, the surface tension f_{st}=F_{ca}. Thus, the Navier–Stokes equations can be expressed as follows[3]:

$$\begin{cases} \rho\frac{\partial\boldsymbol{u}}{\partial t}+\rho\boldsymbol{u}\cdot\nabla\boldsymbol{u}=-\nabla p+\eta\nabla^2\boldsymbol{u}+\frac{1}{3}\eta\nabla(\nabla\cdot\boldsymbol{u})+f_e+f_{st} \\ \frac{\partial(\rho\boldsymbol{u})}{\partial t}+\nabla\cdot(\rho\boldsymbol{u})=0 \end{cases} \tag{4}$$

where the density ρ and viscosity η are smooth functions that depend on the order parameters and satisfy in the i–phase, i.e., $\rho=\rho_1\varphi_1+\rho_2\varphi_2+\rho_3\varphi_3$, $\eta=\eta_1\varphi_1+\eta_2\varphi_2+\eta_3\varphi_3$. In addition, p is the fluidic pressure, f_e is the electrostatic force, and f_{st} is the surface tension at the air–polymer interface or the polymer–polymer interface.

In the growth process, both soft material and rigid material are both considered as the pure dielectric polymer; thus, the conductivity of fluids can be ignored in the numerical simulations. Here, the distribution of electric field inside the numerical domain can be depicted as follows[4]:

$$\nabla\cdot(\varepsilon\boldsymbol{E})=0 \quad \varepsilon=\varepsilon_0\varepsilon_r \tag{5}$$

wherein ε is the permittivity of fluids, ε_0 is the permittivity of vacuum, ε_r is the relative permittivity of air and polymer, and $\boldsymbol{E}$ is the electric field in the numerical domain ($\boldsymbol{E}=-\nabla V$ with V representing

electric potential). Similar to the parameters in Navier–Stokes equation, the parameter of ε_r is also depended on the order parameters, i.e., $\varepsilon_r=\varepsilon_1\varphi_1+\varepsilon_2\varphi_2+\varepsilon_3\varphi_3$. Once an external electric field is applied on the fluids, the Maxwell stress tensor would be generated in the air and polymer with the expression of [5]

$$T_e = \varepsilon \boldsymbol{EE} - \frac{1}{2}E^2\left[\varepsilon - \rho(\frac{\partial \varepsilon}{\partial \rho})\right]\boldsymbol{I} \tag{6}$$

with $\boldsymbol{I}$ is the unit stress tensor. According to the Maxwell stress tensor, we can obtain the electrostatic force acting on the fluids, by

$$f_e = \nabla \cdot T_e = \rho_f \boldsymbol{E} - \frac{1}{2}E^2\nabla\varepsilon + \nabla(\frac{1}{2}E^2\rho\frac{\partial \varepsilon}{\partial \rho}) \tag{7}$$

where ρ_f is the space charge. Here, the experimental materials are considered as pure dielectric, i.e., no space charge, and the relative permittivity of material is not depended on the position. Thus, the expression of electrostatic force can be expressed as

$$f_e = -\frac{1}{2}E^2\nabla\varepsilon \tag{8}$$

It can be seen that the electrostatic force of f_e is concentrated on the interface, and would become nearly zero as the position away from the interface. This can be attributed to the gradient of permittivity of used materials. Furthermore, the electrostatic force can also be expressed in the form of surface force of P_e as[6]:

$$P_e = -\frac{1}{2}\varepsilon_0\varepsilon_i(\varepsilon_i - \varepsilon_j)E_i^2 \tag{9}$$

It can be further expressed in the form of liner force as $F_l = \int_S P_e dl$. Based on the liner formulation, we can obtain the electrowetting contact angle via coupling Lippmann–Young equation, by[7]

$$\cos(\theta) = \cos(\theta_0) + \frac{F_l}{\xi} \tag{10}$$

with θ standing for electrowetting contact angle, θ_0 representing natural contact angle and ξ for surface tension coefficient.

The iteration of this coupled problem is carried out by the finite element method in commercial software of COMSOL Multiphysics, in which the governing equations described in the preceding are defined explicitly and symbolically and then automatically solved by the Galerkin approach. Once the order parameter φ is obtained, the moving liquid−liquid interface can be easily identified by simply color–mapping the order parameter, providing a visualization of the bilayer film deformation step by step.

For flat bilayer film, the geometry values of initial polymer are listed as (Supplementary Fig. 18a): thickness of rigid polymer (h_{rp}) of 20 μm, thickness of soft polymer (h_{sp}) of 20 μm and thickness of air (h_{ap}) of 20 μm. In addition, for prepatterned case, the geometry values of initial bottom polymeric structure are listed as (Supplementary Fig. 18b): width (w) of 70 μm, height (h) of 70 μm, the separation (w_s) between pillars of 70 μm, the separation (h_s) between the electrode pair of 135 μm, and the residual layer with thickness (h_r) of 15 μm.

The relevant simulation parameters for both flat and prepatterned bilayer film are shown in Supplementary Table 1, as well as the parameters used in comparison of voltage and permittivity shown in Supplementary Table 2.

Supplementary Note 2. Analysis of effective driving force at the air–polymer interface and polymer–polymer interface versus the polymeric height for prepatterned case

Electrostatic force (f_e) is the driving force in the vertical-growth stage, and surface tension is the main resistive force (f_{st}), so we defined the effective force of $f_{eff} = f_e - f_{st}$ to discuss the rheological behavior (Supplementary Fig. 19). For the effective force at the air–polymer interface, the initial value

is positive because of the flat shape (no surface tension appears). The action of surface tension develops when the top layer grows, resulting in an abrupt decrease in the effective force. As the top layer moves higher and higher, the effective driving force increases larger and larger due to the narrowing air gap until stepping into stage II. For effective force at the polymer–polymer interface, the initial value is negative due to the exceptionally high surface tension, which can further be attributed to the bottom structural shape in stage I (i). Because the sharp geometrical point is vanished by surface tension as the bottom patterned layer grows, the effective force sharply shifts to a positive value. As the bottom polymer height increases, the slope of effective force increases from tiny to large, then to almost constant, until stage II is reached.

Supplementary Note 3. Numerical analysis of grown core–shell structures contacting to and separating from rough surface

To mimic the contact behavior of an adhesive structure on rough surface, cohesive surface model was employed. The adhesive structure was sketched manually and the rough surface was generated from a 3D scanning image of a piece of ground glass. The sketch of core–shell adhesive structure is shown in Supplementary Fig. 20a. The top and bottom lines were assumed to be flat. Backing layer will joint the core–shell structure on top surface during experiments. The length of top and bottom lines is 80 μm and 120 μm, respectively, and the height is 120 μm. To be noted, when sketching the side curves, the curve must be convex rather than concave, at the corner of side curves and top/bottom lines.

Considering the much larger modulus of ground substrate (i.e., rough surface) in comparison with adhesive structure, the ground substrate was considered as rigid body during simulations, and the adhesive structure was set as deformed body. The adhesive structure domain was divided into two

sections, core section and shell section, when the core section was a 100 μm by 50 μm, and the shell section was the rest of structure (Supplementary Fig. 20a). When the simulated adhesive structure was core–shell, material properties of TPU were used for rigid core section, and material properties of silicon rubber were used for soft shell section. For the mushroom-shaped soft structure, material properties of silicon rubber were adopted as the structural material, identical to the soft part of core–shell structure. For the mushroom-shaped rigid structure, material properties of TPU were adopted as the structural material, identical to the rigid part of core–shell structure. For the normal structure, material properties of PDMS were adopted as the structural material, which is usually used to fabricate adhesive structures according to the published literatures[8-10]. In addition, the hyperelastic tensile behavior of polymeric materials were described by Neo–Hookean model, with the model parameters for different materials given in Supplementary Table 3.

The shell section was partitioned into three parts for high–quality elements and affordable computation. A very fine element size, h_{FEM}=0.1 μm, was adopted at the bottom of adhesive structure and the rough surface to capture their contact behavior (Supplementary Fig. 20b). Two element types, CPS3 and CPS4R, were used. For different adhesive structures (consisting of core–shell structure, soft structure, rigid structure and normal structure), they have the identical meshing topology and elements.

The rough surface was fully pinned, and the movement of adhesive structure was controlled by the displacement of top surface along y direction. The top surface of adhesive structure was coupled with a reference point, RP, and a loading applying to the reference point was equivalent to a loading applying to the whole top surface. The increment size was decided automatically by ABAQUS.

The simulation was divided into three stages: approach, contact and separation. During approach

stage, the adhesive structure moves to a position very close but still separated to the rough surface. When the adhesive structure keeps moving downward, its bottom will get in contact with rough surface (contact stage). With the bottom is impeded by the rough surface, the downward movement of top surface compresses the adhesive structure body. For the core–shell, rigid and normal structures, a same maximum value of compressive force is set, and when reached, the top surface stops moving toward the rough surface and the contact stage ends. For the core–shell and soft structures, an identical conformal contact is considered as the criterion to set the preload on adhesive structures. Finally, an upward loading is applied to separate the adhesive structure from the rough surface.

Here, the cohesive zone theory is adopted to describe the dynamic behavior of the adhesive structure contacting to and separating from the target surface[11-13]. In detail, Von der Waals forces exist in contacting area between the bottom and the rough surface, and the adhesive behavior of micropillar and substrate was simulated through cohesive surface model[11,14,15], where the rough surface was selected to be master surface (surface), and the contact region of micropillar was selected to be slave surface (node). The cohesive behavior was described by a traction-separation response, as shown in Supplementary Fig. 21. The traction t is defined by P/A, where P is the applied load, and A is the original area. The relationship between traction t and separation δ is considered to be liner, namely,

$$t = K\delta \quad (11)$$

where K is stiffness. In our simulation, normal stiffness component K_{nn}, and shear stiffness components, K_{ss} and K_{tt}, were uncoupled and all set to be 1 N/mm. Damage of traction-separation response is initiated by the maximum nominal stress criterion, given by[16],

$$\max\left\{\frac{t_n}{t_{nmax}}, \frac{t_s}{t_{smax}}, \frac{t_t}{t_{tmax}}\right\} = 1 \quad (12)$$

where t_n, t_s, t_t are tractions during separation, and t_{nmax}, t_{smax}, t_{tmax} are maximum tractions for the

cohesive contact. In our case, t_{nmax}=0.2 kPa, and t_{smax}= t_{tmax}= 0.3 kPa. When the damage initiation was achieved, a scalar parameter, *D*, is introduced to describe the damage in cohesive surface model and the stiffness was degraded. During the damage evolution, *D* varies from 0 to 1, where *D*=0 means unbroken cohesive surface, and *D*=1 means fully broken cohesive surface. The degraded stiffness is described by

$$t_n = \begin{cases} (1-D)\overline{t}_n, \overline{t}_n > 0 \\ \overline{t}_n \qquad \text{otherwise} \end{cases}$$
$$t_s = (1-D)\overline{t}_s \tag{13}$$
$$t_t = (1-D)\overline{t}_t$$

where $\overline{t}_n$, $\overline{t}_s$ and $\overline{t}_t$ are the stress components predicted by the elastic traction-separation behavior for the current strains without damage.

An effective separation is defined as

$$\delta_m = \sqrt{\langle \delta_n \rangle^2 + \delta_s^2 + \delta_t^2} \tag{14}$$

where δ_n, δ_s, δ_t are separation components on normal and shear directions.

In exponential softening model, damage parameter *D* is calculated as

$$D = 1 - \left\{ \frac{\delta_m^0}{\delta_m^{max}} \right\} \left\{ 1 - \frac{1 - \exp\left(-\alpha\left(\frac{\delta_m^{\max} - \delta_m^0}{\delta_m^f - \delta_m^0}\right)\right)}{1 - \exp(-\alpha)} \right\} \tag{15}$$

where δ_m^0 is the separation when damage initiates, $\delta_m^{\max}$ is the total separation parameter with a set value of 1 μm, and α is exponential softening parameter a set value of 2. To facilitate calculations, a stabilization parameter of 0.001 was adopted. Here, the same cohesive zone parameters were used for core–shell structure, soft structure, rigid structure and normal structure.

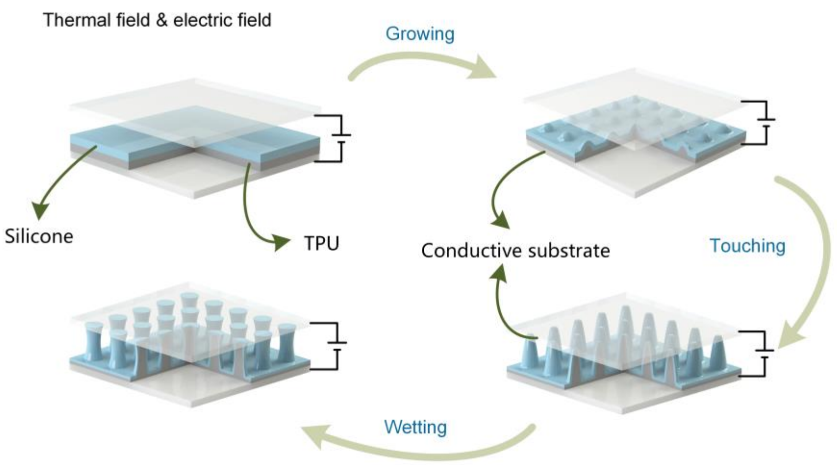

Supplementary Fig. 1 | Schematic of growth process of the mushroom–shaped structures with rigid core–soft shell. Using a flat PDMS mold, a molding technique was used to create the TPU film. The bilayer film was then created by spinning the soft material onto the TPU film's surface. The temperature was increased to 85 °C for the fluidity of TPU and an external electric field applied between two electrodes was applied to the bilayer film. The top layer film will grow vertically to the upper electrode, make contact with the upper electrode, and grow horizontally on the electrode surface as a result of the electrostatic force, creating a mushroom-shaped geometry. A rigid core-soft shell structure results from the bottom layer's simultaneous growth being driven by the electric field and constrained by the top polymer film. We raised the temperature to 90 °C to cure the top polymer when the growing process was complete, and then we decreased it to room temperature to cure the bottom layer. The grown core-shell structures were obtained after we removed the top electrode.

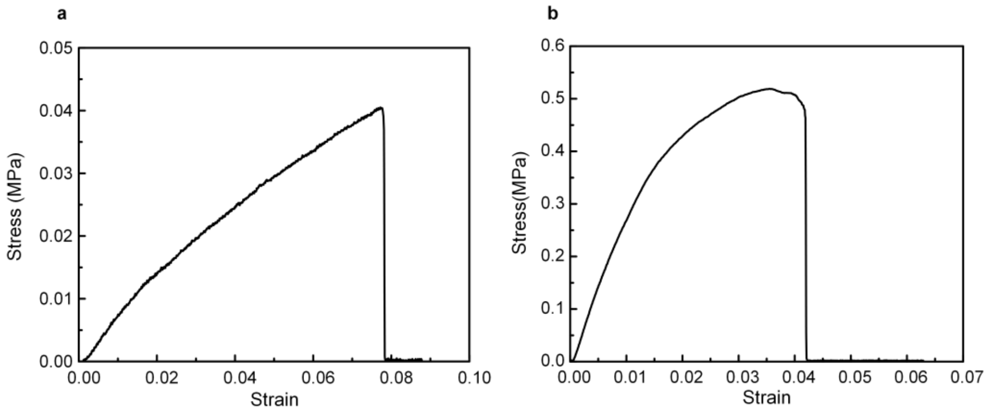

Supplementary Fig. 2 | Stress–strain curves of polymer used in the experiments, consisting of (a) silicone acting as soft material after curved and (b) TPU acting as rigid material after curved. Based on the curves, we can calculate that the elasticity modulus of silicone is roughly 0.5 MPa and the modulus of TPU is roughly 26 MPa. In detail, the testing material was firstly prepared into a sheet with a length of 50 mm, a width of 10 mm and a thickness of 1 mm. Before testing, a triangular notch with a depth of 1 mm was introduced on the middle of both sides of the sample, so that the sample could fracture from there. The sample was then stretched by the tensile test machine, in which the lower end was fixed and the upper end was stretched at a speed of 10 mm/min. Finally, the stress-strain curve of the sample under tensile process was established. A tangent line was then drawn on the initial nearly linear part of the stress-strain curve, and the ratio of stress/strain on the tangent line was the tensile elastic modulus.

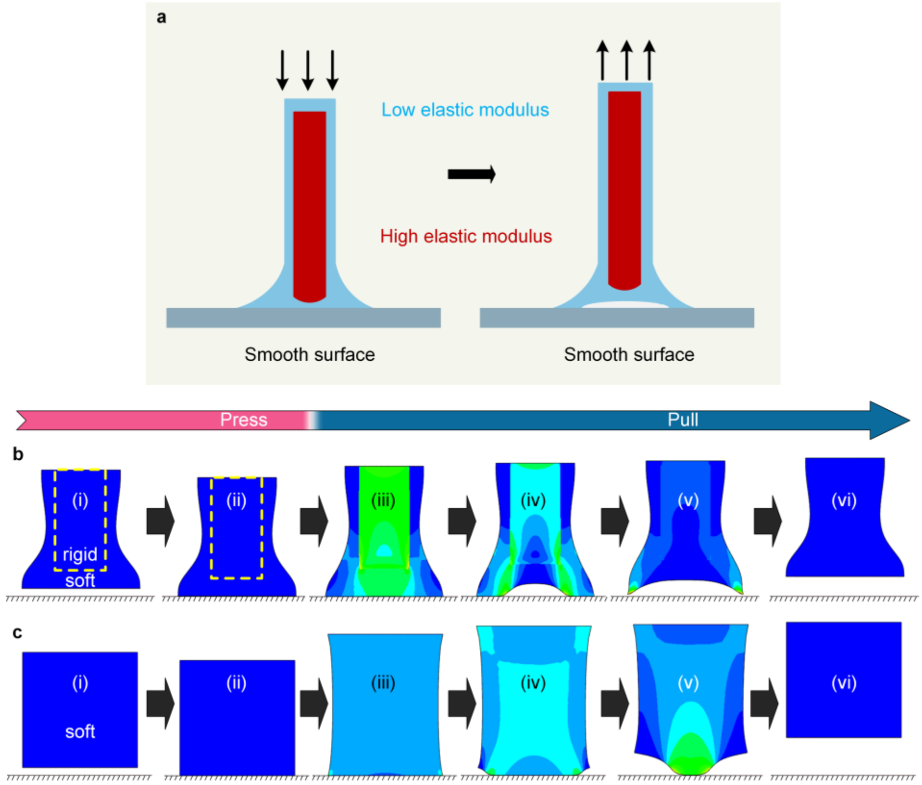

Supplementary Fig. 3 | Demonstration of the crack generated in the central section of the interface between the core-shell structure and the smooth surface. a, Schematic of adhesive behavior for the rigid core–soft shell structure approaching to and separating from a smooth surface. The colors of light blue and crimson represent soft polymer and rigid polymer, respectively. **b, c,** Dynamic behavior of the core–shell structure (**b**), soft structure with the material corresponding to the soft part of the core–shell structure (**c**) when (i) approaching, (ii) contacting, and (iii–vi) separating from the smooth surface. Cloud atlas representing the internal stress. Obviously, owing to the stress distribution, a crack is generated in the central section of the interface for mushroom-shaped core-shell structure; in contrast, the crack is observed at the edge of the adhered interface for micro-pillar with flat cap.

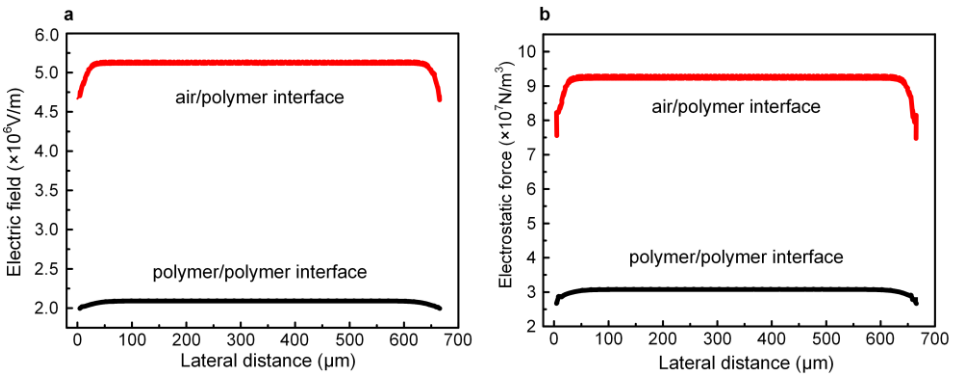

Supplementary Fig. 4 | Distribution of (a) electric field and (b) electrostatic force on the air–polymer interface and polymer–polymer interface at the initial growing stage. Obviously, the distribution of electrostatic force is similar to that of electric field, implying that the electrostatic force acting on the bilayer film is determined by the electric field.

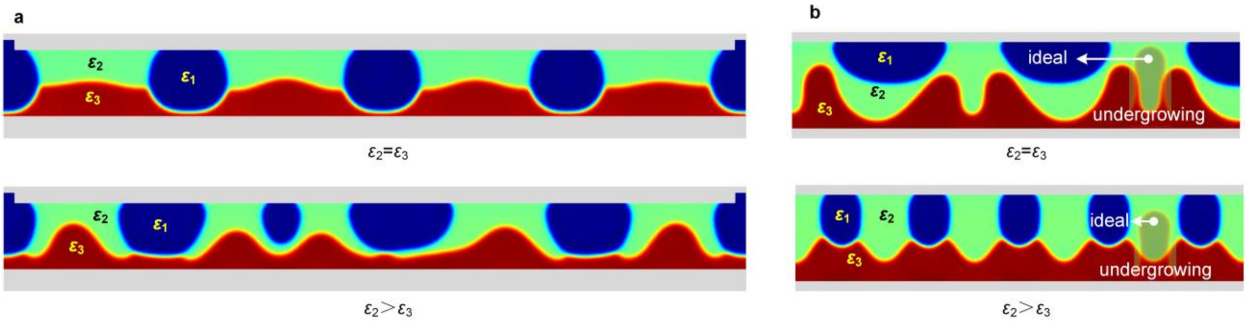

Supplementary Fig. 5 | Polymeric deformation of bilayer film with different relative permittivity. **a**, deformation of bilayer polymer film under an external electric field for flat case. **b**, deformation of bilayer polymer film under an external electric field for prepatterned case. The colors of blue, green and crimson represent air, soft polymer and rigid polymer, respectively.

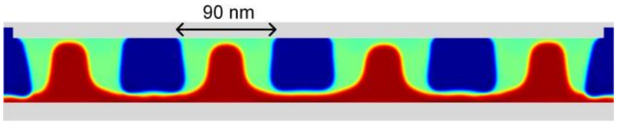

Supplementary Fig. 6 | Grown core–shell structures with a characteristic length of roughly 90 nm from the viewpoint of numerical simulation. The nanoscale structures can be generated via precisely controlling the parallelism between electrode pairs and the geometric variables (consisting of polymeric height, air gap, etc.) at nanoscale. The colors of blue, green and crimson represent air, soft polymer and rigid polymer, respectively.

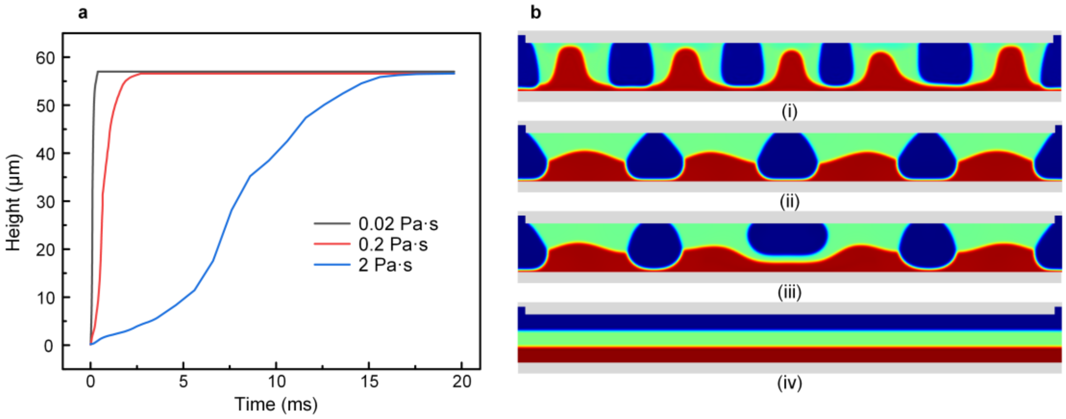

Supplementary Fig. 7 | Grown core–shell structures with different viscosity coefficient. a, Variation of structure height versus the processing time under different viscosity coefficient. **b**, Generated structures under different viscosity coefficient with (i)~(iv) representing 0.02 Pa·s, 0.2 Pa·s, 2 Pa·s, and 20 Pa·s, respectively. The colors of blue, green and crimson represent air, soft polymer and rigid polymer, respectively.

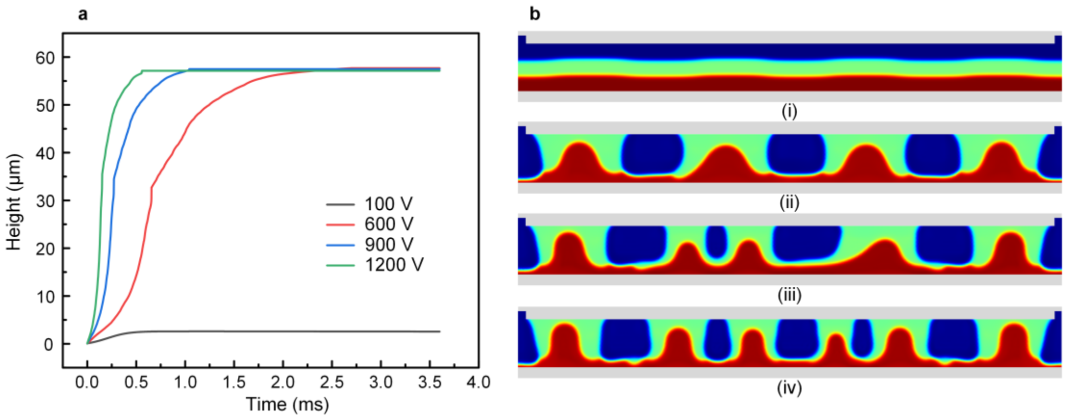

Supplementary Fig. 8 | Grown core–shell structures with different external voltage. a, Variation of structure height versus the processing time under different voltage. **b**, Generated structures under different applied voltage with (i)~(iv) representing 100 V, 600 V, 900 V, and 1200 V, respectively. The colors of blue, green and crimson represent air, soft polymer and rigid polymer, respectively.

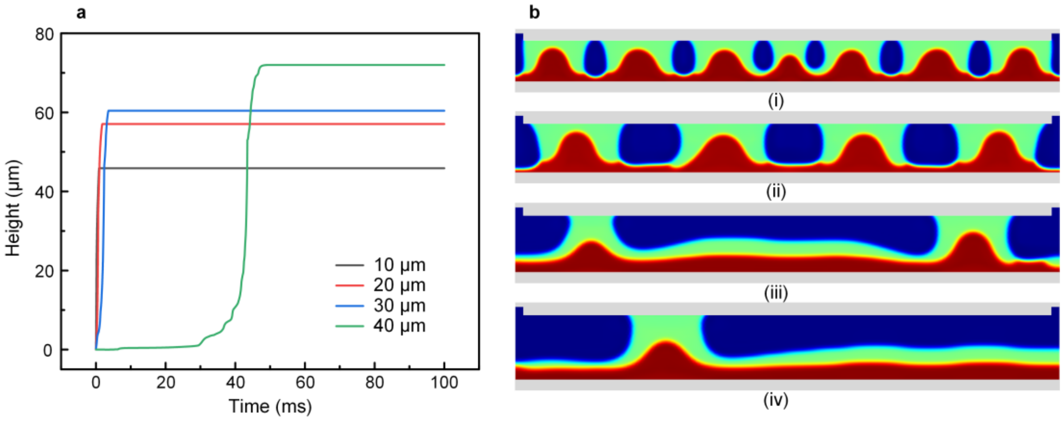

Supplementary Fig. 9 | Grown core–shell structures with different air gap between the upper electrode and the soft polymer. a, Variation of structure height versus the processing time under different air gap thickness. **b**, Generated structures under different air gap thickness with (i)~(iv) representing 10 μm, 20 μm, 30 μm, and 40 μm, respectively. The colors of blue, green and crimson represent air, soft polymer and rigid polymer, respectively.

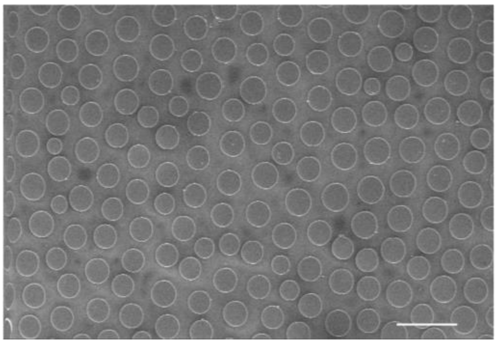

Supplementary Fig. 10 | Grown core–shell structures from the top view. Owing to no restriction on the electric field, short-range order can be seen in the distribution of grown structures. In other words, a small region has a regular diameter or periodicity, but a large area has a weaker regularity. The scale bar is 600 μm.

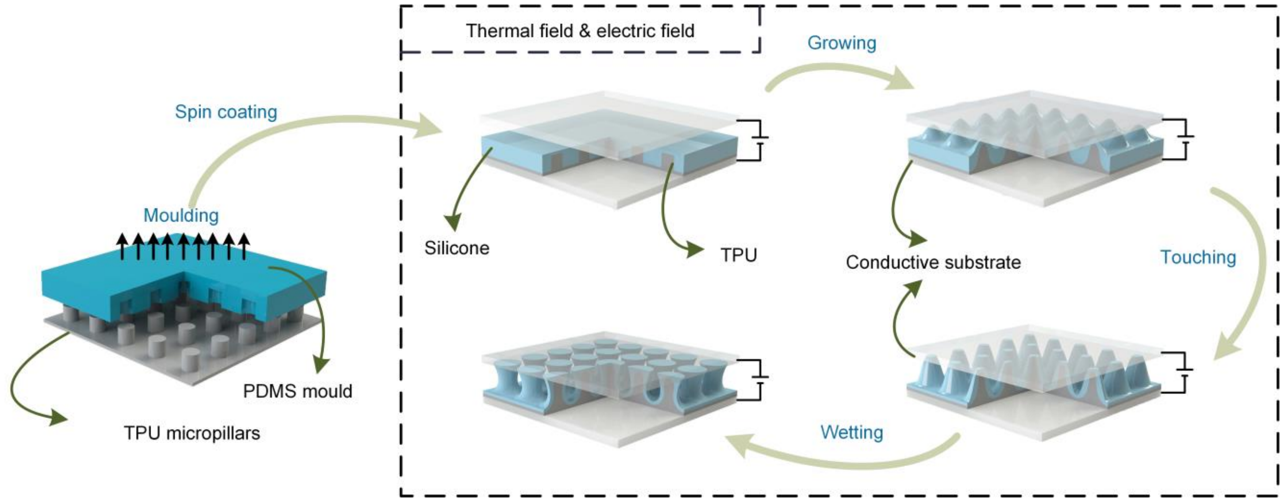

Supplementary Fig. 11 | Schematic of growth process of the mushroom–shaped structures with rigid core–soft shell for prepatterned case. The process is similar to that of flat bilayer film except for the prepatterns on the bottom layer obtained firstly. Here, the TPU structures with low aspect ratio was obtained via moulding method using a structured PDMS mold, which acted as the initial pattern. The following procedure is identical to that of flat bilayer film. In this case, the pillars are simultaneously growing upwards to the electrode rather than the sequence from the edge to the center.

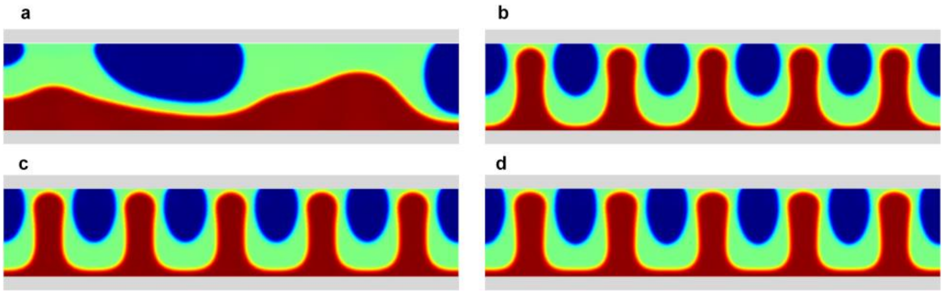

Supplementary Fig. 12 | Grown core–shell structures under different external voltage for prepatterned case with (a) 500 V, (b) 1000 V, (c) 1500 V, and (d) 2000 V. Obviously, the core-shell structures cannot be generated with a low voltage of 500 V. As the voltage is increased to 1000 V, the anticipated structure can be generated with the regularity corresponding to the initial pattern. If the voltage is further increased to 1500 V and even 2000 V, the regularity can still be maintained for the prepatterned case, which is different from the flat case. This phenomenon can be attributed to the spatially modulated electric field incurred by the prepattern. The colors of blue, green and crimson represent air, soft polymer and rigid polymer, respectively.

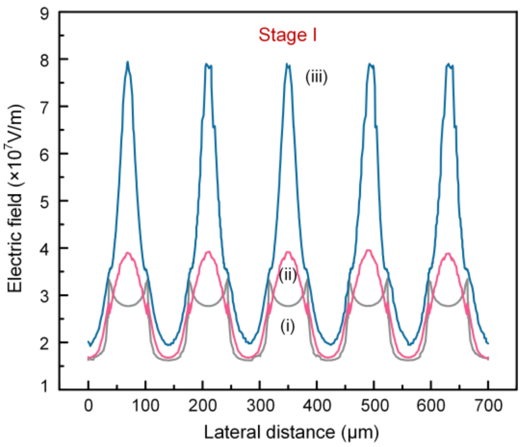

Supplementary Fig. 13 | Electric field distribution on the polymer–polymer interface at different snapshot of stage I for the prepatterned case. The evolution of electric field on the polymer–polymer interface demonstrates a relationship of positive feedback with the pillar height until touching the upper electrode.

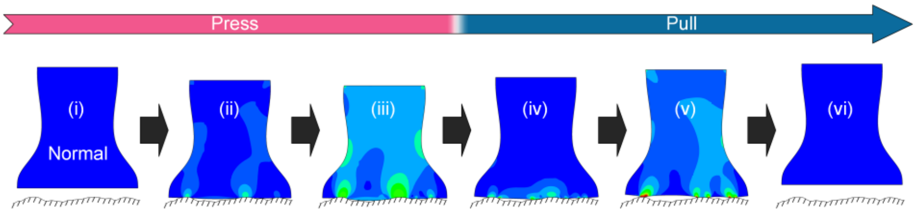

Supplementary Fig. 14 | Dynamic behavior of the normal structure on rough surface. (i) Approaching to the rough surface. (ii) – (iii) Contacting the rough surface. (iv) – (vi) Separating from the rough surface. Cloud atlas representing the internal stress.

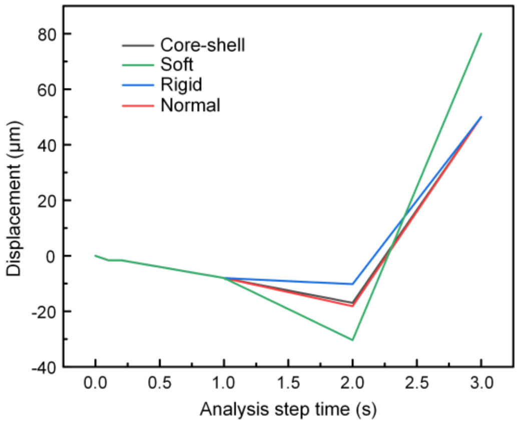

Supplementary Fig. 15 | Variation of displacement versus analysis step time in the numerical simulations. In the simulation of adhesive structure contacting to and separating from testing surfaces, the control variable is displacement. In detail, at the time of 0 ~ 1 s, the adhesive structure approached to the testing surface without any contact. Starting at 1 s, the adhesive structure began to contact the testing surface, and the contact area became larger and larger until it reached 2 s. After 2 s, the adhesive structure began to detach from the testing surface until the adhesive structure was completely separated from the testing surface. Here, Core–shell, Soft, Rigid and Normal represent mushroom-shaped structure with a rigid core and a soft shell, mushroom-shape structure with soft material, mushroom-shape structure with rigid material, and mushroom-shaped structure with common elastic material, respectively.

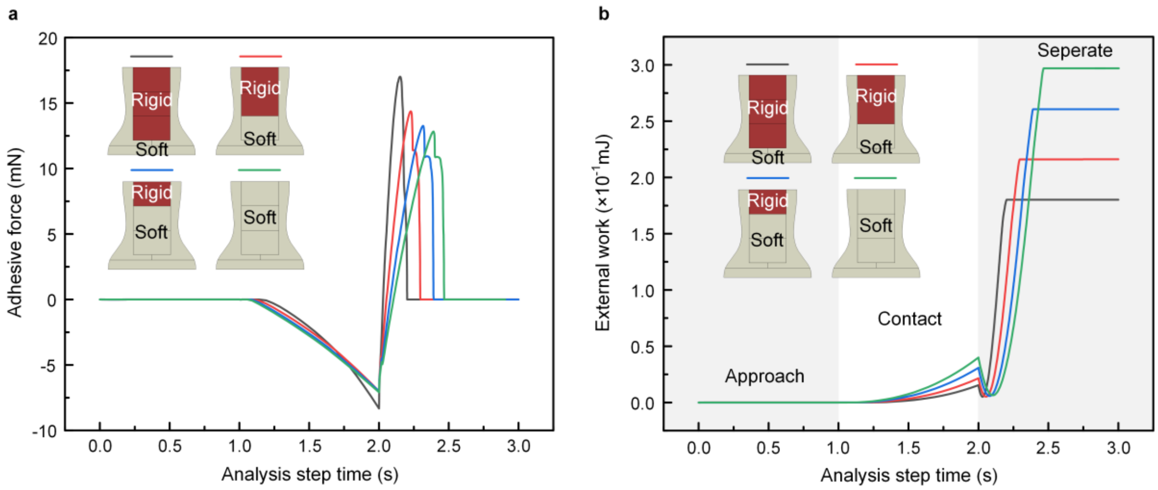

Supplementary Fig. 16 | Influence of structural stiffness on the adhesion with the varied stiffness achieved by adjusting the height of rigid core pillar. a, Adhesive force as a function of the process time for different adhesive structures. **b,** Work of attachment/detachment as a function of the process time for different adhesive structures.

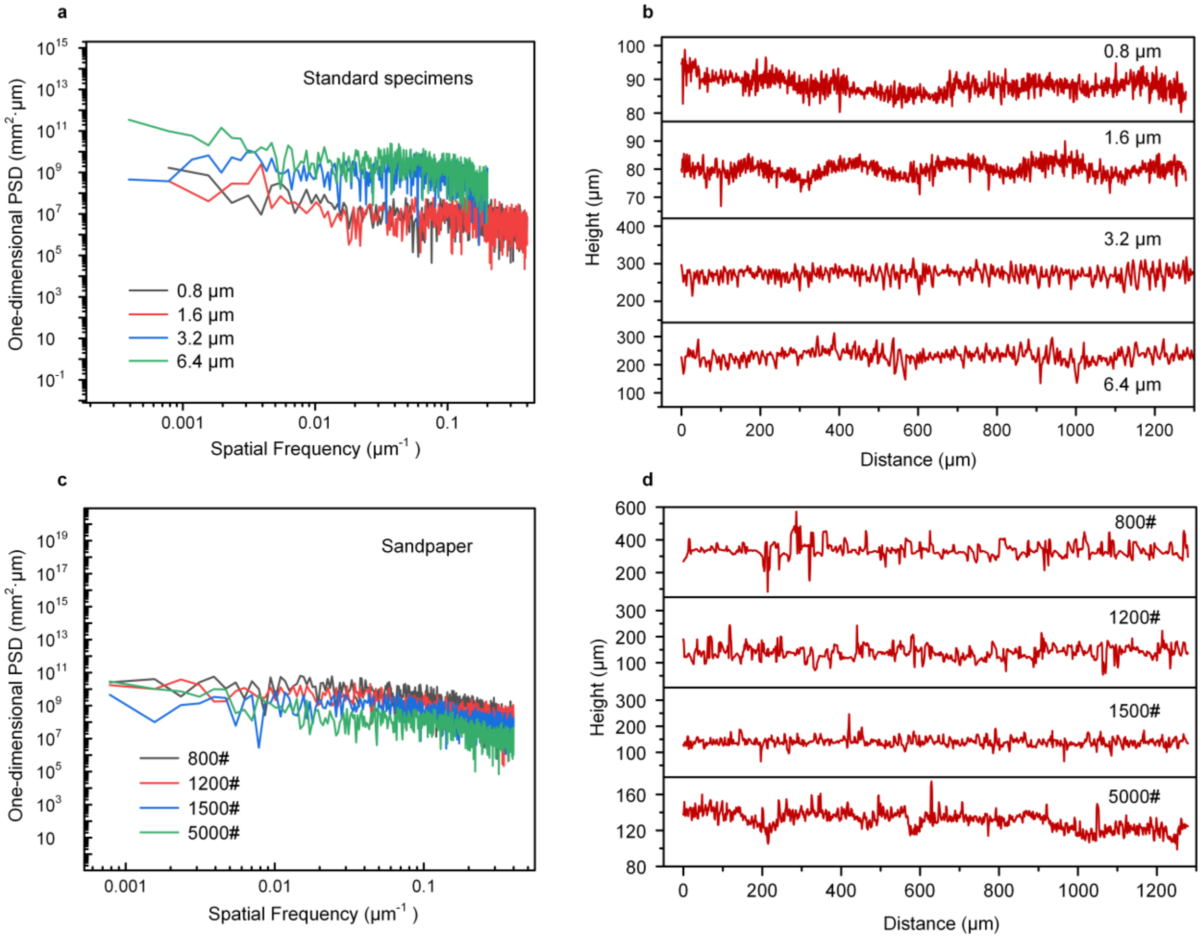

Supplementary Fig. 17 | Characteristics of rough surfaces used for testing the adhesive force of different adhesive structures. a, Power special density (PSD) of standard specimens. **b**, Surface morphology of standards specimens obtained by laser scanning confocal microscope. **c**, Power special density of sandpapers. **d**, Surface morphology of sandpapers obtained by laser scanning confocal microscope.

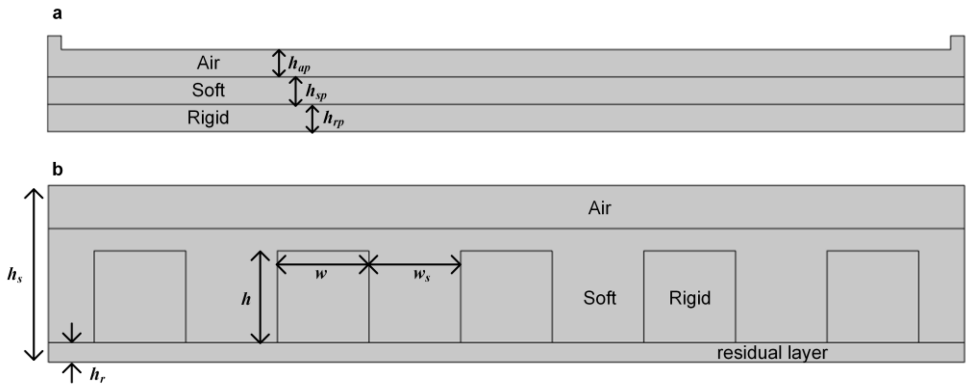

Supplementary Fig. 18 | Schematic of initial geometry for (a) flat bilayer film and (b) prepatterned bilayer film under an exerted electric field.

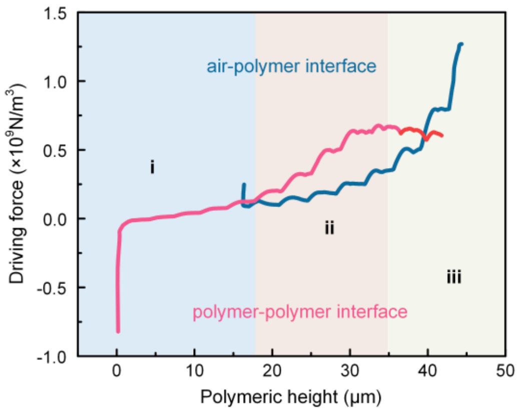

Supplementary Fig. 19 | Variation of effective driving force at the air–polymer interface and polymer–polymer interface versus the polymeric height.

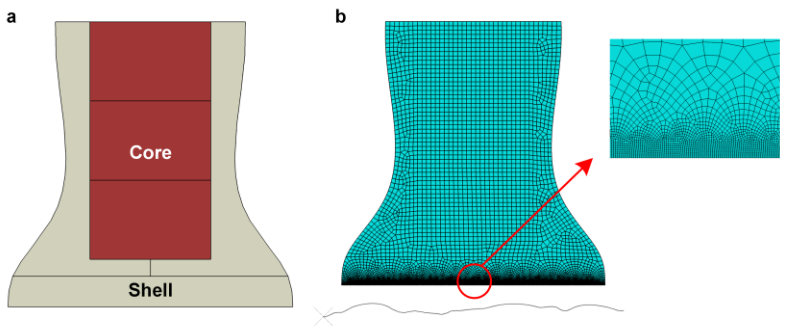

Supplementary Fig. 20 | Schematic of (a) core–shell structure and (b) meshed core–shell structure. As the comparison, the mushroom–shaped homogeneous structure (consisting of soft, rigid and normal ones) is similar to that of mushroom–shaped one with rigid core–soft shell.

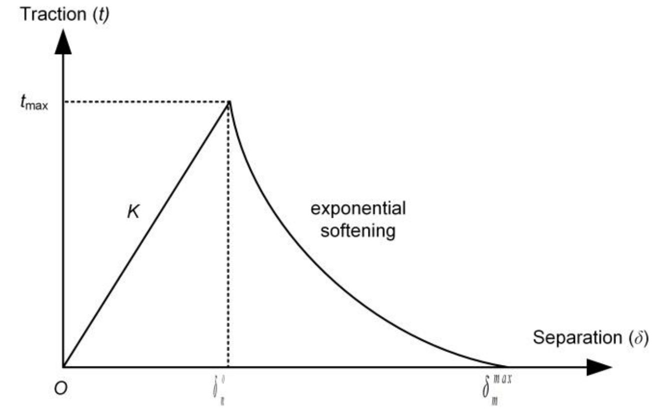

Supplementary Fig. 21 | Sketch of traction-separation response for cohesive behavior of adhesive structures in the numerical simulations.

Supplementary Table 1. Parameters used in numerical simulation of growth process

Simulation parameters	Ternary fluids	Value
Mass density	Air	1 kg/m^3
Mass density	Soft polymer	1000 kg/m^3
Mass density	Rigid polymer	1200 kg/m^3
Relative permittivity	Air	1
Relative permittivity	Soft polymer	5
Relative permittivity	Rigid polymer	10
Viscosity	Air	1×10^{-4} Pa·s
Viscosity	Soft polymer	0.2 Pa·s
Viscosity	Rigid polymer	0.2 Pa·s
Surface tension coefficient	Air–soft polymer interface	0.03 N/m
Surface tension coefficient	Air–rigid polymer interface	0.03 N/m
Surface tension coefficient	Soft polymer–rigid polymer interface	0.01 N/m
Voltage	Flat film	600 V
Voltage	Structured film	1000 V

Supplementary Table 2. Parameters used in numerical comparisons of growth process

Simulation parameters	Comparison	Value
Relative permittivity (Flat)	Equaling	$\varepsilon_2=\varepsilon_3=5$
Relative permittivity (Flat)	Reversal	$\varepsilon_2=20$, $\varepsilon_3=5$
Relative permittivity (Prepatterned)	Equaling	$\varepsilon_2=\varepsilon_3=5$
Relative permittivity (Prepatterned)	Reversal	$\varepsilon_2=10$, $\varepsilon_3=5$

Supplementary Table 3. Hyperelastic models and material properties for mechanical analysis of core-shell structures

Materials	Hyperelastic models	Parameters
Normal material (PDMS)	Neo–Hookean	C_{10}=0.65, D_1=0.25
Soft material (Silicon rubber)	Neo–Hookean	C_{10}=0.15, D_1=0.25
Rigid material (TPU)	Neo–Hookean	C_{10}=12.5, D_1=0.25

Supplementary References:

1 Boyer, F., Lapuerta, C., Minjeaud, S., Piar, B. & Quintard, M. Cahn–Hilliard/Navier–Stokes Model for the Simulation of Three-Phase Flows. *Transp. Porous Media* **82**, 463-483 (2009).

2 Boyer, F. & Lapuerta, C. Study of a three component Cahn-Hilliard flow model. *ESAIM: Math. Modell. Numer. Anal.* **40**, 653-687 (2006).

3 Junseok, K. & Lowengrub, J. Phase field modeling and simulation of three-phase flows. *Interface. Free. Bound.* **7**, 435-466 (2005).

4 Jackson, J. D. & Levitt, L. C. Classical Electrodynamics. *Phys. Today* **15**, 62-62 (1962).

5 J. R. Melcher & Taylor, G. I. Electrohydrodynamics: A review of the role of interfacial shear stresses. *Annu. Rev. Fluid Mech.* **1**, 111-146 (1969).

6 Fan, S. K., Hsieh, T. H. & Lin, D. Y. General digital microfluidic platform manipulating dielectric and conductive droplets by dielectrophoresis and electrowetting. *Lab Chip* **9**, 1236-1242 (2009).

7 Kang, K. H. How Electrostatic Fields Change Contact Angle in Electrowetting. *Langmuir* **18**, 10318-10322 (2002).

8 Wang, Y., Hu, H., Shao, J. & Ding, Y. Fabrication of well-defined mushroom-shaped structures for biomimetic dry adhesive by conventional photolithography and molding. *ACS Appl. Mater. Interfaces* **6**, 2213-2218 (2014).

9 Sameoto, D. & Menon, C. A low-cost, high-yield fabrication method for producing optimized biomimetic dry adhesives. *J. Micromech. Microeng.* **19**, 115002 (2009).

10 Ara´nzazu, del, C., Christian, G. & Eduard, A. Contact Shape Controls Adhesion of Bioinspired Fibrillar Surfaces. *Langmuir* **23**, 10235-10243 (2007).

11 Zhang, X., Wang, Y., Hensel René & Eduard Arzt. A Design Strategy for Mushroom-Shaped Microfibrils with Optimized Dry Adhesion: Experiments and Finite Element Analyses. *J. Appl. Mech.* **88**, 031015 (2021).

12 Koloor, S, S, R., Rahimian-Koloor, S, M. , Karimzadeh, A., Hamdi, M., Michal. & Tamin, M. N. Nano-Level Damage Characterization of Graphene/Polymer Cohesive Interface under Tensile Separation. *Polymers* **11**, 1435 (2019).

13 Jiang, H., Yiru, R. & Gao, B. Research on the progressive damage model and trigger geometry of composite waved beam to improve crashworthiness. *Thin Wall Struct.* **119** 531–543 (2017).

14 Autumn, K. *et al.* Adhesive force of a single gecko foot-hair. *Nature* **405**, 681-685 (2000).

15 Autumn, K. *et al.* Evidence for van der Waals adhesion in gecko setae. *Proc. Natl. Acad. Sci. U. S. A.* **991**, 12252-12256 (2002).

16 Rahul-Kumar, P., Jagota, A., Bennison, S.J. & Saigal, S. Interfacial failures in a compressive shear strength test of glass/polymer laminates. *Int. J. Solids Struct.* **37**, 7281-7305 (2000).
