# Supplementary material for: Core–shell dry adhesives for rough surfaces via electrically responsive self-growing strategy
Source: Nat Commun. 2022 Dec 10;13:7659. doi: 10.1038/s41467-022-35436-6 (PMC9741600; doi:10.1038/s41467-022-35436-6)
Supplement: Supplementary file 2 — Description of Additional Supplementary Files [file 41467_2022_35436_MOESM2_ESM.pdf]

Description of Additional Supplementary Files

File Name: Supplementary Movie 1
Description: Numerically dynamic growing evolution of bilayer polymer film under an external electric field for flat case.

File Name: Supplementary Movie 2
Description: Numerically dynamic growing evolution of bilayer polymer film under an external electric field for prepatterned case.

File Name: Supplementary Movie 3
Description: Numerically dynamic contacting–separating behavior of mushroom–shaped structure with rigid core–soft shell.

File Name: Supplementary Movie 4
Description: Numerically dynamic contacting–separating behavior of mushroom–shaped structure with soft material.

File Name: Supplementary Movie 5
Description: Numerically dynamic contacting–separating behavior of mushroom–shaped structure with rigid material.

File Name: Supplementary Movie 6
Description: Numerically dynamic contacting–separating behavior of mushroom–shaped structure with normal material.
